# Programmable cell–cell adhesion in synthetic yeast communities for improved bioproduction

**DOI:** 10.1038/s41589-025-02081-1

**Published:** 2026-01-05

**Authors:** Haohong Chen, Huadong Peng, Tom Ellis, Rodrigo Ledesma-Amaro

**Affiliations:** 1 Imperial College Centre for Synthetic Biology, Imperial College London, London, UK; 2 Bezos Centre for Sustainable Protein, Imperial College London, London, UK; 3 Engineering Biology Microbial Food Hub, Imperial College London, London, UK; 4 Department of Bioengineering, Imperial College London, London, UK; 5 School of Food Science and Engineering, South China University of Technology, Guangzhou, China; 6 Australian Institute for Bioengineering and Nanotechnology, The University of Queensland, Brisbane, Queensland Australia; 7 ARC Centre of Excellence in Synthetic Biology, The University of Queensland, Brisbane, Queensland Australia; 8 Food and Beverage Accelerator (FaBA), The University of Queensland, Brisbane, Queensland Australia

**Keywords:** Metabolic engineering, Industrial microbiology

## Abstract

In multicellular systems, engineering-controlled cell–cell adhesion and metabolic interdependence are vital for developing complex functionalities. This study introduces a yeast synthetic toolbox for modular cell–cell adhesion and cocultures, aiming to overcome the limitations of existing approaches that lack genetic specificity and control. First, a model yeast strain 007Δ is created with seven main flocculation and agglutination genes removed, providing a clean background for synthetic adhesion systems. Then, three distinct adhesion pair systems—Strategy 1, Strategy 2.1 and Strategy 2.2—are established involving yeast flocculation and agglutination proteins and yeast surface display systems. In addition, a quantitative assessment is conducted on the adhesive specificity and strength, alongside the capability of synthetic adhesion to generate patterns. Finally, we successfully demonstrate enhanced bioproduction of the high-value food antioxidant, resveratrol, utilizing synthetic cocultures coupled with cell adhesion systems. We anticipate that this toolkit will emerge as a valuable resource for diverse applications in synthetic biology and biomanufacturing.

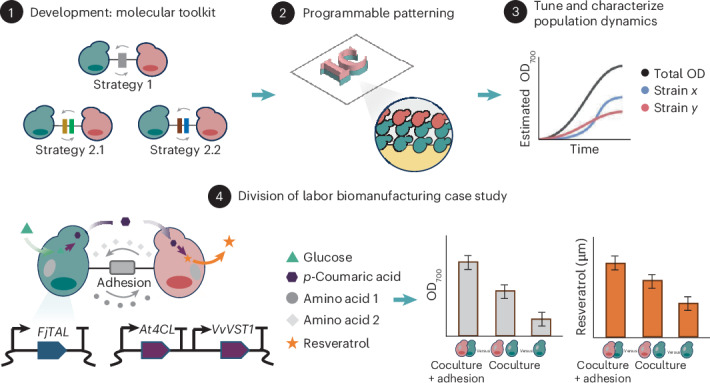

## Abstract (web summary)

Programming microbial interactions can enhance biomanufacturing. Here, the authors develop a synthetic yeast toolbox that programs cell–cell adhesion and cross-feeding, enabling spatial patterning coupled with division of labor to boost production of the high-value antioxidant resveratrol.

## Main

Microbial communities are ubiquitous in nature and have diverse applications in the bioeconomy^1,2^, including the bioproduction of biochemicals^3,4^, biomedicines^5,6^, biofuels^7^ and biomaterials^8^ from inexpensive carbon sources. Modular coculture engineering, an emerging approach in synthetic biology, involves designing synthetic microbial communities with specialized cell populations to optimize specific metabolic pathways, mimicking the division of labor observed in nature^2,4^. This approach has gained substantial interest because of its potential to enhance the production of valuable compounds and create more efficient bioprocesses compared to single-strain systems^4,5,9^. However, engineering stable microbial communities remains a challenge because of the complex cell–cell interactions and metabolic dynamics between different species and their environment^2,10^. To address this, researchers are increasingly applying synthetic microbial communities to study and manipulate multicellular interactions, aiming to develop robust, multifunctional communities for different biotechnological applications.

The study and engineering of multicellularity could allow a wide range of morphologies and spatial patterns at various scales and across different cell types, like those found in multicellular organisms. The distinctive capabilities of multicellular arrangements include the separation of intermediates in complex metabolic pathways for improved bioproduction^9,11,12^ and the programming of structured living materials^8,13^. Formation of these complex structures and arrangements is primarily driven by intercellular signaling mechanisms and external stimuli. These factors work together to regulate gene expression, which in turn influences the activation of key components such as adhesion molecules, ligand receptors and synthetic polymers in microbial communities^2,14,15^.

Cell–cell adhesion mediated by surface molecules represents a promising approach for creating three-dimensional, multicellular aggregates. This approach has important implications for understanding the natural development of biofilms, higher organisms and microbial communities^16–18^. In particular, genetically encoded synthetic adhesion molecules, comprising an outer membrane tether, and pairs of adhesins and nanobodies, offer precise control over modular cell–cell adhesion in microbial communities^15,18^. This has been successfully demonstrated in *Escherichia coli*^15^, which has yielded multicellular aggregates exhibiting diverse structures such as mesh-like patterns, fibrous formations and spheroid morphologies. Notably, we have recently extended application of this toolkit to yeast, facilitating the creation of cell aggregation patterns and multicellular logic circuits in *Saccharomyces cerevisiae*^19^. These aggregates and patterns provide invaluable insights into cooperative dynamics and evolutionary processes, serving as powerful tools for engineering complex multicomponent metabolic pathways and materials^9,18^. Although synthetic scaffolds have been explored as an alternative approach to enhance cell adhesion and community formation^15,17,20,21^, they often present limitations such as limited control over specificity, direct association with signaling events or a nongenetic basis that can be diluted by growth or chemical modification^22–24^. Thus, the genetically encoded adhesion toolkit could provide a more controllable and potentially more robust approach to engineering cell adhesion. Despite this potential, the application of cell–cell adhesion tools in multicellular engineering, especially for constructing synthetic communities with bioproduction capacity, remains largely unexplored.

Metabolic interdependencies, such as cross-feeding, are common in natural microbial communities^4,10,12,25^. Coauxotrophic strains often form mutually dependent communities, in which the survival of each member relies on the supplementation of missing metabolites by other consortium members. Coculture strategies typically improve production performance by reducing the metabolic burden of microorganisms or minimizing inhibitory feedback^4,12,25^. Our previous efforts have successfully proven that both cross-feeding and division of labor improve bioproduction in synthetic yeast communities^4,12,25^. Building on these findings, we now aim to investigate whether spatial control via cell adhesion molecules is also beneficial for bioproduction.

Photoswitchable adhesion proteins and cross-feeding have been shown to control the formation and behavior of multicellular *E. coli* communities^26^, and a mathematical model has suggested that cell adhesion confers a fitness advantage to the *E. coli* in a stirred cross-feeding coculture with *S. cerevisiae*^14^. Although these two studies have explored the combined impact of cross-feeding and adhesion in synthetic communities, such as *E. coli*–*E. coli* (in vivo)^26^ and *E. coli*–*S. cerevisiae* cocultures (in silico)^14^, the potential of this combination remains largely unknown in yeast. It is therefore important to investigate the combined effects of adhesion and cross-feeding, or the trifecta of adhesion, cross-feeding and division of labor, on bioproduction in synthetic yeast communities.

In this work, to address these limitations and facilitate controlled multicellular self-assembly in yeast cocultures, we develop a toolbox for engineering cell–cell adhesion and cross-feeding interactions. First, we create a yeast strain with major flocculation and agglutination genes removed, followed by the design of three adhesion pair systems involving flocculation, agglutination, surface display and adhesion components. Then, we quantitatively examine the specificity and strength of synthetic cell–cell adhesion, both with and without cross-feeding interactions, assessing their impact on pattern formation and cell growth. Finally, utilizing this toolbox, we successfully enhance the production of the high-value aromatic compound resveratrol through synergistic manipulation of cell–cell adhesion, cross-feeding and division of labor in yeast cocultures.

## Results

### Cell–cell adhesion facilitates two-dimensional pattern in synthetic cocultures

**Fig. 1:**
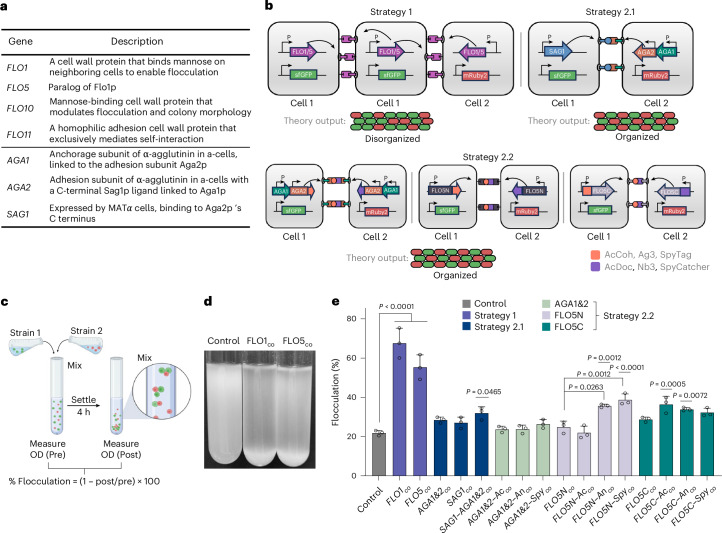
Synthetic adhesion toolkit for multicellular pattern assembly and cell–cell adhesion tuning library. **a**, The model strain (007Δ) features seven gene deletions: *FLO1*, *FLO5*, *FLO10* and *FLO11* to prevent flocculation, and *AGA1*, *AGA2* and *SAG1* to eliminate the agglutinin system^30–34^. **b**, Spatial programming in a synthetic yeast coculture is achieved by expressing specific adhesion pairs in each strain. Strategy 1—yeast flocculation principle: one strain expressed the *FLO1* gene, while the other expressed the *FLO5* gene. Strategy 2.1—yeast agglutinin system utilization: cell 1 was engineered to express the *SAG1* gene, whereas cell 2 expressed both *AGA1* and *AGA2* genes. Strategy 2.2—yeast surface display system application: in cell 1, the *AcCoh*, *Ag3* and *SpyTag* genes were introduced, while in cell 2, the corresponding genes *AcDoc*, *Nb3* and *SpyCatcher* were introduced. **c**, Intercellular binding strength and specificity are quantified through an aggregation assay based on OD_600nm_ measurements. **d**, Visible aggregation and settling in 1:1 mixtures of cells expressing *FLO1* or *FLO5*. **e**, Quantification of flocculation percentages across three adhesion pair systems, comparing aggregating mixtures to the adhesin-free control. *n* = 3 biologically independent samples and data are presented as mean ± s.d. Statistical analysis was executed using Prism 9.5.0 (GraphPad) software with one-way analysis of variance (ANOVA), followed by Tukey’s post hoc test and *P* values were noted (FLO1_co_:control, *P* < 0.0001; FLO5_co_:control, *P* < 0.0001; SAG1–AGA1&2_co_:control, *P* = 0.0465; FLO5N–An_co_:control, *P* = 0.0012; FLO5N–Spy_co_:control, *P* < 0.0001; FLO5C–Ac_co_:control, *P* = 0.0005; FLO5C–Ac_co_:control, *P* = 0.0072; FLO5N–An_co_:FLO5N_co_, *P* = 0.0263; FLO5N–Spy_co_: FLO5N_co_, *P* = 0.0012). Schematic in **c** created with BioRender.com. Source data

**Extended Data Fig. 1:**
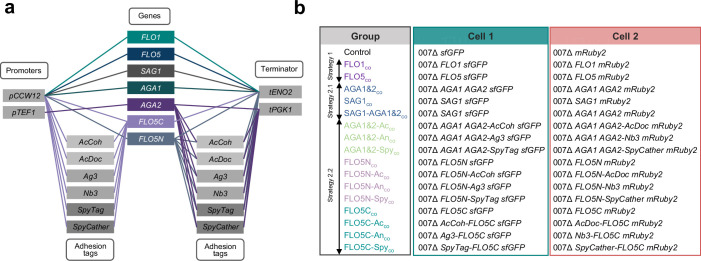
Construction of adhesion gene modules and genotypic design of surface display strains. **a**, Schematic of flocculation and agglutinin gene expression constructs and adhesion pair refactoring. Surface display vector architecture was shown in Supplementary Fig. 1. **b**, The phenotype of each member and the strain combination for surface display are listed in the table. 007Δ: BY4741 *flo1*Δ *flo5*Δ *flo10*Δ *flo11*Δ *aga1*Δ *aga2*Δ *sag1*Δ.

Cell–cell adhesion, mediated by flocculation and agglutination, is essential for cell survival, cooperation and communication^27–29^. Flocculation refers to the reversible aggregation of yeast cells into clusters through *FLO* gene family proteins. Flo1p, Flo5p and Flo10p primarily contribute to general flocculation, binding to mannose residues present on neighboring cells^30–32^, while Flo11p additionally influences biofilm formation and invasive growth through increased cell-surface adhesion properties^29,33^. Agglutination induces cell clumping via the specific surface adhesins the α-agglutinin heterodimer Aga1p–Aga2p and the α-agglutinin Sag1p, which mediate mating-type recognition and binding. Aga1p anchors Aga2p onto the cell surface, allowing Aga2p to specifically recognize and bind to Sag1p on other yeast cells, thus facilitating precise cell–cell adhesion required for mating^34^. Here we explored flocculation, agglutination or surface display systems with adhesion components to promote cell adhesion in yeast (Fig. 1a,b). First, we constructed the model yeast strain 007Δ by knocking out seven main flocculation and agglutination genes. *FLO1*, *FLO5*, *FLO10* and *FLO11* were deleted to eliminate flocculation, while *AGA1*, *AGA2* and *SAG1* were removed to disrupt agglutination (Fig. 1a). Second, we established one nonspecific adhesion pair system named Strategy 1, and two specific adhesion pair systems named Strategy 2.1 and Strategy 2.2. Strategy 1 involving flocculation genes *FLO1* (or *FLO5*) enables nonspecific cell–cell adhesion through flocculation mechanisms, with Flo1p and Flo5p being homologous proteins that bind to mannose chains on the surface of other cells^30,32^. Strategy 2.1 introduces the mating protein Sag1p in cell 1 to target the dimeric Aga1p–Aga2p (AGA1&2) in cell 2 for agglutination, enabling specific adhesion^34,35^. Strategy 2.2 involves using the N-terminal (FLO5N) and C-terminal (FLO5C) regions of Flo5p, along with AGA1&2, as surface display platforms for orthogonal combinations with specific adhesion proteins and their targeted ligands such as SpyTag–SpyCatcher (Spy), Ag3–Nb3 (An) and AcCoh–AcDoc (Ac)^15,28^ (Fig. 1b and Extended Data Fig. 1). Both the surface display platforms of FLO5C and AGA1&2 are constructed through cell-surface glycosylphosphatidylinositol-anchor domains in the protein as scaffolds, while FLO5N achieves surface display by binding to mannose chains on the cell surface through the secreted protein form^28,32^. Components used for Strategy 2.2 were recently described as part of a modular toolkit for orthogonal and programmable yeast multicellularity^19^.

Following the methodology of Veelders et al.^32^, we mixed pairwise combinations of the engineered yeast cell types (cell 1, marked with sfGFP; cell 2, marked with mRuby2), as depicted in Fig. 1c, and measured the flocculation ratio by comparing the optical density (OD) at intermediate heights before and after 4 h of static incubation (pre and post OD). The control (007Δ *sfGFP*^Cell 1^–007Δ *mRuby2*^Cell 2^) remained turbid, whereas the coculture overexpressing *FLO1* and *FLO5* genes showed notable cell sedimentation after 4 h (Fig. 1d). The highest flocculation ratios were observed in Strategy 1, reaching 63.8% for the FLO1_co_ coculture and 52.1% for the FLO5_co_ coculture (Fig. 1e), indicating that *FLO1* and *FLO5* overexpression substantially enhanced flocculation. Even under continuous shaking, cell aggregation persisted (Supplementary Fig. 2), suggesting that the increased interaction due to *FLO1* and *FLO5* expression drives flocculation. Moreover, the SAG1–AGA1&2_co_ coculture in Strategy 2.1 showed significantly higher flocculation (31.4%) than the control (*P* = 0.0465) (Fig. 1e), highlighting the importance of *AGA1*, *AGA2* and *SAG1* overexpression in yeast agglutination. Interestingly, the AGA1&2 surface display coculture groups in Strategy 2.2 alone did not substantially differ from the control.

In addition, we also observed the cocultures under confocal microscopy to visualize pairwise adhesion between cell 1 (expressing sfGFP) and cell 2 (expressing mRuby2) at an OD ratio of 1:1 across Strategy 1, Strategy 2.1 and Strategy 2.2 (Supplementary Fig. 3a–c). Adhesion patterns were categorized into five distinct types based on aggregation forms: multicellular with red and green, multicellular with red or green, tricellular–bicellular with red and green, tricellular–bicellular with red or green, and single cell (Supplementary Fig. 3d). In the control, both cell types, often in mother–daughter cell states^36^, predominantly existed as single cells or small clusters of up to three cells. Strategy 1 showed notable aggregation of both cell types, forming large clusters with few single cells. Although aggregation was also observed in Strategy 2.1 and Strategy 2.2, the cluster sizes were smaller. In Strategy 2.2, the AGA1&2 surface display system with adhesion tags exhibited the highest frequency of mixed-cell clusters, followed by the FLO5C system, while the FLO5N system showed the least.

**Fig. 2:**
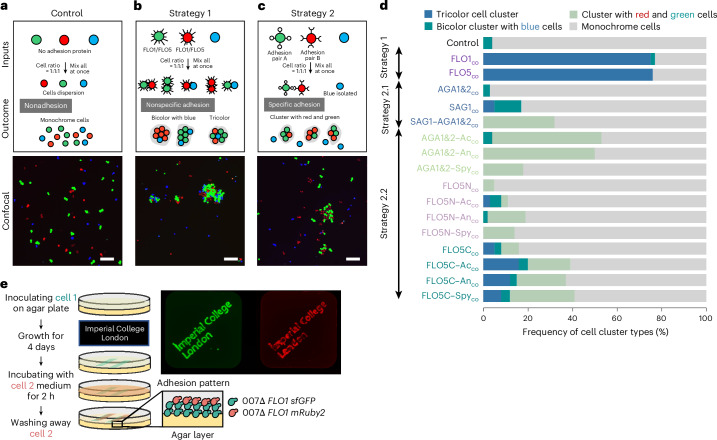
Rational design and evaluation of complex multicellular patterns through specific synthetic adhesion in yeast. **a–c**, Schematic representation and confocal microscopy images demonstrating adhesion specificity using a nonadhesion reference cell 3 (blue). Figures present representative fluorescent images from experiments that were independently repeated at least three times with similar outcomes. **a**, In the control strain with seven adhesion genes deleted (*FLO1*, *FLO5*, *FLO10*, *FLO11*, *AGA1*, *AGA2*, *SAG1*), all cell types remain dispersed. Microscopy images (strains): 007Δ *sfGFP* (green cells), 007Δ *mRuby2* (red cells), 007Δ *mTagBFP2* (blue cells). **b**, Expression of *FLO1* or *FLO5* in cell 1 and cell 2 results in nonspecific adhesion to cell 3. Microscopy images (strains): 007Δ *FLO1 sfGFP* (green cells), 007Δ *FLO1 mRuby2* (red cells), 007Δ *mTagBFP2* (blue cells). **c**, Specific adhesion pairs (Strategy 2) mediate adhesion between cell 1 and cell 2, but not to cell 3. Microscopy images (strains): 007Δ *SAG1 sfGFP* (green cells), 007Δ *AGA1 AGA2 mRuby2* (red cells), 007Δ *mTagBFP2* (blue cells). Strategy 2.1 was selected as an example of Strategy 2. Strategy 2.2 showed a similar pattern and can be found in Supplementary Fig. 3e. **d**, Quantification of the percentage of each cell cluster type after introducing cell 3 (blue) across various adhesion systems (Strategy 1, Strategy 2.1 and Strategy 2.2). Tricolor cell clusters are cell clusters containing all three fluorescent colors (green, red and blue); bicolor clusters with blue are cell clusters containing two colors, one of which is blue; cluster with red and green cells are cell clusters containing only red and green cells; and monochrome cells are single-colored cells. Quantification was determined by calculating the ratio of each cell type to the total cell count, derived from six confocal images obtained from confocal sections of *n* = 3 biologically independent replicates. **e**, Typhoon biomolecular imager analysis of 2D pattern formation using engineered yeast cells expressing *FLO1*. Cell 2 (007Δ *FLO1 mRuby2*) adheres to cell 1 (007Δ *FLO1 sfGFP*), replicating the pattern of cell 1. Scale bars, 50 µm. Source data

Not all *FLO* genes exhibit nonspecific adhesion properties. For instance, *FLO11* encodes a homophilic adhesion protein that specifically mediates self-interaction—it specifically interacts with itself after expression^29^. By contrast, Flo1p and Flo5p proteins interact with the cell wall and exhibit more general adhesive properties, making them nonspecific ‘sticky’ factors^30,34^. To assess adhesion specificity, a nonadhesion reference cell (blue cell 3, 007Δ *mTagBFP2*) was introduced into cocultures across all adhesion pair systems (Fig. 2a–c). Cell 1, cell 2 and cell 3 were mixed at an OD ratio of 1:1:1. Subsequent analysis of cell composition identified four distinct categories: tricolor cells cluster (clusters containing cell 1, cell 2 and cell 3), bicolor cluster with blue cells (clusters composed of cell 1 and cell 3 or cell 2 and cell 3), cluster with red and green cells (clusters containing only cell 1 and cell 2) and monochrome cells (clusters consisting of a single cell type). If cells are able to adhere to cell 3, which lacks adhesion capability, it indicates that their adhesion is nonspecific. In the coculture control, all cell types remained separate, with ‘monochrome cells’ accounting for 96%, indicating no adhesion (Fig. 2d). However, Strategy 1 exhibited nonspecific clustering, with more than 74% of all three cell types aggregating together, while the specific adhesion category ‘cluster with red and green cells’ was almost absent. By contrast, Strategy 2.1 and Strategy 2.2 displayed specific adhesion, with clusters forming exclusively between cell 1 and cell 2, while cell 3 remained dispersed, resulting in a predominance of ‘monochrome cells’. In Strategy 2.2, AGA1&2–Ac_co_ and AGA1&2–An_co_ exhibited higher specificity adhesion ability, with ‘cluster with red and green cells’ over 49%. Interestingly, in the Strategy 2.2 FLO5C surface display system, more than 5% of clusters were tricolor, indicating a degree of nonspecific binding alongside the expected specific adhesion. This may be attributed to residual weak nonspecific adhesion activity in FLO5C. Veelders et al. proposed that the BC region in FLO5C also contributes to cell–cell adhesion^32^. Moreover, length variation of repetitive tandem repeats in the B region in FLO5C was reported to alter flocculation and biofilm formation of yeast^37^. FLO5N does not directly bind to the cell surface via glycosylphosphatidylinositol, but rather attaches to the cell surface mannose chains as a secreted protein^32,38^, which limits the occurrence of flocculation.

Engineered cell–cell adhesion combined with coculture strategies offers an alternative approach to formulating controlled two-dimensional (2D) patterns, which can have applications in cellular engineering and biotechnology^39^. Our sedimentation experiments revealed Flo1p as a potent driver of cell aggregation. Flo1p is also reported to enable yeast cells to invade agar and adhere strongly^29^. We therefore investigated the use of *FLO1*-overexpressing cocultures (FLO1_co_) in combination with cell–cell adhesion for 2D pattern formation, which was imaged with an Amersham Typhoon scanner. An ‘Imperial College London’ pattern was formed on a synthetic minimal (SM) agar plate using *FLO1*- and *sfGFP*-expressing cell 1 as input signal. After 4 days of incubation, a second layer of *FLO1*- and *mRuby2*-expressing cell 2 was applied over the whole plate, and the plate was gently washed with SM medium to test adhesion stability. In contrast to the control cocultures of 007Δ_co_ (007Δ *sfGFP*^Cell 1^–007Δ *mRuby2*^Cell 2^) and BY4741_co_ (BY4741 *sfGFP*^Cell 1^–BY4741 *mRuby2*^Cell 2^), the FLO1_co_ coculture (007Δ *FLO1 sfGFP*^Cell 1^–007Δ *FLO1 mRuby2*^Cell 2^) demonstrated strong adherence of cell 2 to cell 1, forming a stable overlay (Fig. 2e and Supplementary Fig. 4). This confirms that *FLO1*-mediated yeast cell–cell adhesion can be harnessed to aggregate diverse cell types at specific locations, enabling the creation of complex cellular structures with potential applications in tissue engineering^8,39^.

### Cross-feeding and adhesion control patterning and growth

**Fig. 3:**
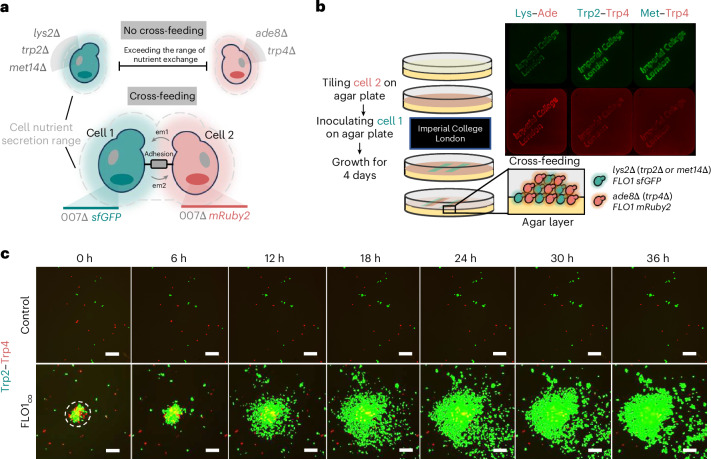
Engineered cross-feeding and cell–cell adhesion enable controlled 2D pattern formation in synthetic yeast cocultures. **a**, Diagram of two-member cross-feeding cocultures (Lys–Ade, Trp2–Trp4 and Met–Trp4 (refs. ^4,12,40^)). Each member is auxotrophic to one exchanged metabolite and overproduced another, with cell 1 expressing *sfGFP* (green) and cell 2 expressing *mRuby2* (red). **b**, Three proven synthetic cross-feeding coculture pairs were used to assess their ability to form complex 2D patterns. Pattern formation occurs only where cell 1 (*lys2*Δ (*trp2*Δ or *met14*Δ) *FLO1 sfGFP*) and cell 2 (*ade8*Δ (or *trp4*Δ) *FLO1 mRuby2*) are coinoculated. **c**, Time-lapse confocal microscopy visualization of synthetic cocultures exhibiting cross-feeding and adhesion. Self-assembly of multicellular aggregates promotes cell growth in these cocultures. White circles indicate rapidly growing cell clusters. Time-lapse images (strains): *trp2*Δ *FLO1 sfGFP*, *trp4*Δ *FLO1 mRuby2*. Trp2–Trp4 was selected as a representative example, with additional coculture pairs shown in Extended Data Fig. 2 and Supplementary Videos 1–6. Samples with *FLO1* expression, where red and green cells formed clusters, were selected as the representative slides. Quantification was performed by averaging three confocal slices from *n* = 3 biologically independent samples, and the results are presented in Supplementary Fig. 7. em1, exchanged metabolite 1; em2, exchanged metabolite 2. Scale bars, 50 µm.

To minimize interference from yeast’s native flocculation and agglutination, we systematically deleted seven related genes (namely *FLO1*, *FLO5*, *SAG1*, *AGA1*, *AGA2*, *FLO10* and *FLO11*) in five auxotrophic yeast strains—lysine auxotrophs (*lys2*Δ), adenine auxotrophs (*ade8*Δ), tryptophan auxotrophs (*trp2*Δ, *trp4*Δ) and methionine auxotrophs (*met14*Δ). Unless otherwise specified, the cross-feeding strains are all based on 007Δ. These strains are auxotrophic for essential amino acids or nucleotides: lysine (Lys), adenine (Ade), tryptophan (Trp) and methionine (Met), respectively (Fig. 3a, Supplementary Fig. 5a and Supplementary Table 1). Importantly, specific combinations of these engineered strains have been previously proved to form three distinct two-member model (cell 1–cell 2) cross-feeding coculture systems: Lys–Ade, Trp2–Trp4 and Met–Trp4 (refs. ^12,40^). Two auxotrophic cells, cell 1 and cell 2, must be present simultaneously to form cross-feeding for growth (Supplementary Fig. 5b). This suggests that the cross-feeding functional characteristic can also be utilized to achieve cell programmability. To investigate pattern formation in cross-feeding cocultures, we first spread a lawn of 200 μl of *ade8*Δ or *trp4*Δ cell 2 (expressing mRuby2) onto SM agar. We then inoculated *lys2*Δ, *trp2*Δ or *met14*Δ cell 1 (expressing sfGFP) onto the plate in a predefined pattern as input signal (Fig. 3b). After 4 days of incubation, imaging revealed that cell 2 growth (red fluorescence) as the output signal occurred exclusively where cell 1 had been inoculated. Conversely, areas that did not receive cell 1 inoculation displayed no growth of cell 2. The distant cell 2, far from the cross-feeding nutrient center, was unable to benefit from cross-feeding. This outcome indicates the specificity and strict spatial restriction of the interaction between these cell types and highlights the effectiveness of this method in directing cell growth in spatially controlled patterns. Besides, pattern formation also occurred in nonauxotrophic (cell 1)–auxotrophic (cell 2) pairs (Supplementary Fig. 6). However, when both cells are prototrophic or if cell 1 is auxotrophic and cell 2 is prototrophic, the pattern does not form, and the protocol generates a lawn of cells.

**Extended Data Fig. 2:**
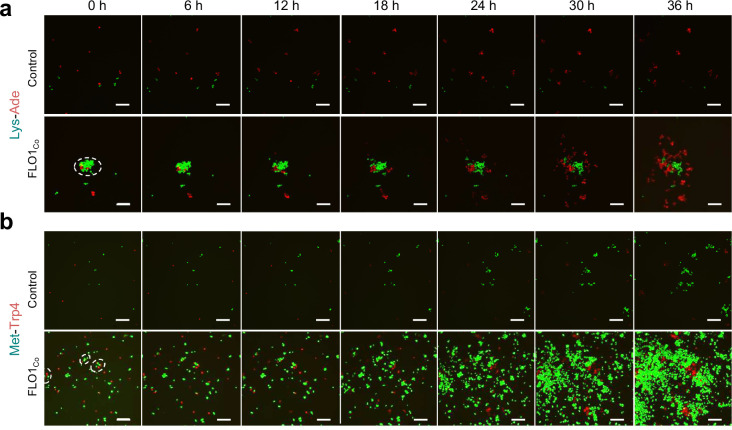
Time-lapse confocal microscopy visualization of Lys-Ade and Met-Trp4 synthetic cocultures. **a**, Time-lapse images (Strains): *lys2*Δ *FLO1 sfGFP*, *ade8*Δ *FLO1 mRuby2*. **b**, Time-lapse images (Strains): *met14*Δ *FLO1 sfGFP*, *trp4*Δ *FLO1 mRuby2*. White circles indicate rapidly growing cell clusters. Samples with *FLO1* expression, where red and green cells formed clusters, were selected as the representative slides. Quantification was performed by averaging three confocal slices from *n* = 3 biologically independent samples, and the results are presented in Supplementary Fig. 7. Scale bar, 50 µm.

To explore the effects of adhesion and cross-feeding on growth and population distribution in yeast cocultures, three auxotrophic systems (Lys–Ade, Trp2–Trp4 and Met–Trp4) were combined with *FLO1* gene through time-lapse confocal microscopy to observe experimental setups. Compared to the control, *FLO1* expression induced notable aggregation of two yeast cell types at 0 h, and accelerated their proliferation (Fig. 3c, Extended Data Fig. 2, Supplementary Fig. 7 and Supplementary Videos 1–8). Specifically, in the cross-feeding cocultures, the auxotrophic strains (red cell 2 *ade8*Δ, *met14*Δ or green cell 1 *trp2*Δ) showed higher cell densities, linked to enhanced growth. Conversely, cross-feeding cocultures that did not overexpress the *FLO1* gene remained dispersed and displayed lower cell densities, and, therefore, slower growth (Supplementary Fig. 7b and Supplementary Videos 1–6). This difference in growth patterns highlights the crucial role of both adhesion and cross-feeding in modulating cellular proliferation and demonstrates that adhesion can improve syntrophy and growth.

**Fig. 4:**
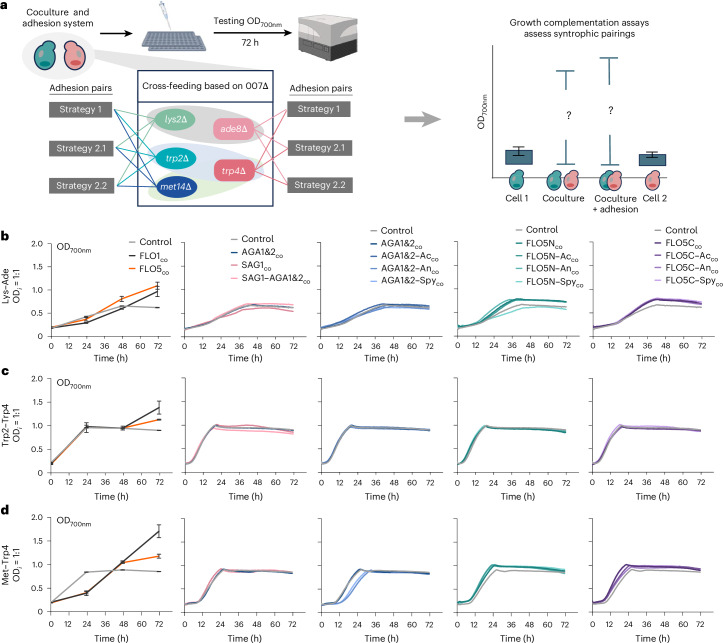
Engineered cross-feeding and cell–cell adhesion enable controlled growth dynamics in synthetic yeast cocultures. **a**, Diagram of cell growth assay using a microplate reader and the design of cross-feeding cocultures with different surface display architectures. Three auxotrophic systems (Lys–Ade, Trp2–Trp4 and Met–Trp4) were combined with 16 adhesion pairs across three adhesion pair strategies (Strategy 1, Strategy 2.1 and Strategy 2.2). **b**–**d**, Growth curves (OD_700nm_) of the three cross-feeding cocultures over 72 h, each inoculated at a 1:1 initial ratio and incorporating adhesion systems: Lys–Ade (**b**), Trp2–Trp4 (**c**) and Met–Trp4 (**d**). *n* = 3 biologically independent samples and data are presented as mean ± s.d. Data for other initial ratios (1:10 and 10:1) are shown in Supplementary Figs. 8, 10 and 12. Schematic in **a** created with BioRender.com. Source data

**Extended Data Fig. 3:**
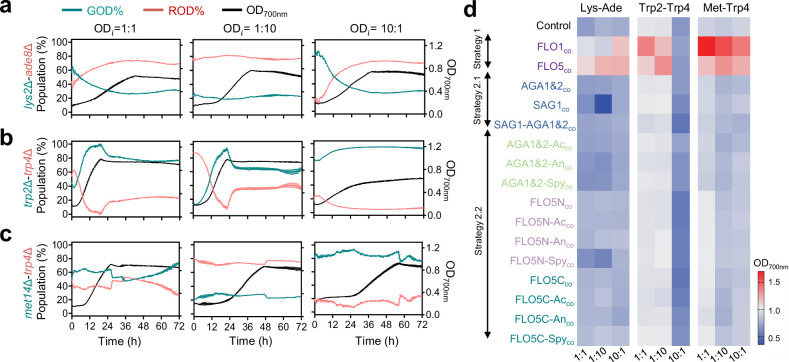
Growth dynamics and cell proportions in synthetic yeast cocultures. **a-c**, Growth curves of the cross-feeding coculture pairs and estimated growth of individual population percentages at different initial cell ratios over 72 h (**a**, *lys2*Δ–*ade8*Δ; **b**, *trp2*Δ–*trp4*Δ; **c**, *met14*Δ–*trp4*Δ). Cell 1: *lys2*Δ (*trp2*Δ or *met14*Δ) *sfGFP*; Cell 2: *ade8*Δ (or *trp4*Δ) *mRuby2*. OD_700nm_ represents the total coculture density. GOD%: The proportion of GFP intensity-converted OD relative to the total OD_700nm_; ROD%: The proportion of mRuby2 intensity-converted OD relative to the total OD_700nm_. Data for cocultures with different adhesion pairs are shown in Supplementary Fig. 9, 11, 13. Standard curves for converting fluorescence intensities (GOD and ROD) to OD values are provided in Supplementary Table 2. *n* = 3 biologically independent samples and data are presented as mean ( ± s.d.). **d**, Heatmap of maximal OD_700nm_ values within 72 h for each coculture with 16 adhesion pairs, across initial ratios of 1:1, 1:10, and 10:1. *n* = 3 biologically independent samples and data are presented as mean. Source data

Controlling population dynamics in synthetic cocultures, particularly those with regulated cell–cell adhesion, is crucial for achieving desired cellular outcomes and improving our understanding of complex biological interactions^2^. Our research next focused on regulating population sizes in modular cross-feeding cocultures by incorporating three cell–cell adhesion strategies (Fig. 4 and Extended Data Fig. 3). Previous work identified the initial population ratio as a key factor influencing coculture dynamics^4^. We observed distinct growth patterns in the Lys*–*Ade coculture under different initial OD ratios (Fig. 4b and Supplementary Fig. 8). At both 1:1 and 1:10 ratios, cell 2 (*ade8*Δ) dominated the coculture, reaching a stable plateau (Extended Data Fig. 3a). However, at a 10:1 ratio, cell 1 (*lys2*Δ) initially dominated but was eventually overtaken by cell 2. Introducing adhesion pairs did not substantially affect cell 2 dominance, except in the Strategy 2.2 FLO5C system coculture at a 1:1 ratio, where it reduced cell 2 dominance (Supplementary Fig. 9d). *FLO1* and *FLO5* expression enhanced the maximal OD regardless of the initial ratio (Supplementary Fig. 8b). In the Trp2*–*Trp4 coculture, cell 1 (*trp2*Δ) exhibited a growth advantage over cell 2 (*trp4*Δ) across all initial OD ratios (Extended Data Fig. 3b and Supplementary Figs. 10 and 11). *FLO1* and *FLO5* expression equally enhanced the maximal OD at 1:1 and 1:10 ratios (Fig. 4c and Supplementary Fig. 10b). The Met*–*Trp4 coculture showed fluctuating proportions, with cell 1 dominance at a 10:1 ratio and cell 2 dominance at a 1:10 ratio (Extended Data Fig. 3c and Supplementary Figs. 12 and 13). *FLO1* and *FLO5* expression also increased the maximal OD in this coculture (Extended Data Fig. 3c,d and Supplementary Fig. 12b). Therefore, initial population ratios and the introduction of adhesion pairs substantially influence the growth dynamics and final population composition of cross-feeding cocultures. *FLO1* and *FLO5* expression consistently enhanced overall growth (Extended Data Fig. 3d), whereas the specific impact of adhesion pairs on population dominance varied depending on the coculture system and initial conditions. Further details on the growth dynamics of cross-feeding cocultures with cell adhesion under various initial conditions are provided in Supplementary Figs. 8–13.

### Synthetic yeast cocultures boost resveratrol production

**Fig. 5:**
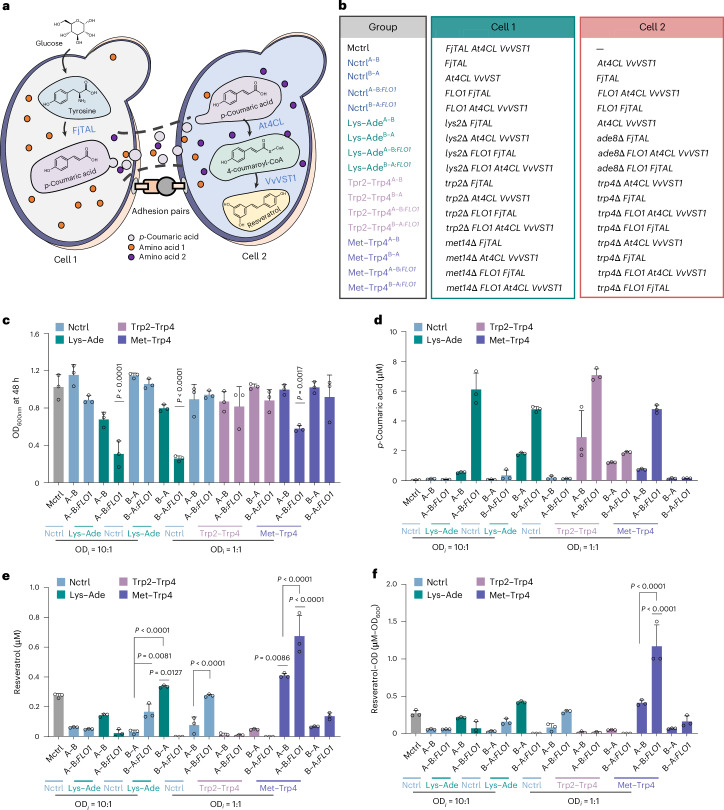
Division of labor, cross-feeding and cell–cell adhesion improve resveratrol production in synthetic yeast cocultures. **a**, Schematic representation of the de novo resveratrol synthesis pathway in yeast and division of labor in cross-feeding yeast cocultures. **b**, Table summarizing strain combinations for three cocultures and their corresponding surface display systems. Target gene abbreviations are used to label monoculture controls (each strain) and two-member cocultures. **c**–**f**, At 48 h in SM medium, OD_600nm_ (**c**), *p*-coumaric acid (**d**), resveratrol concentrations (**e**) and resveratrol–OD_600nm_ (**f**) were measured and analyzed for three coculture pairs and their corresponding adhesion systems. ‘A–B’ refers to module A being present in cell 1 and module B in cell 2, whereas ‘B–A’ signifies module B in cell 1 and module A in cell 2. *n* = 3 biologically independent samples and data are presented as mean ± s.d. Statistical analysis was executed using Prism 9.5.0 (GraphPad) software with one-way ANOVA, followed by Tukey’s post hoc test and *P* values were noted. In **c**, at OD_*i*_ = 10:1, Lys–Ade^A–B:*FLO1*^:Mctrl, *P* < 0.0001; Lys–Ade^B–A:*FLO1*^:Mctrl, *P* < 0.0001; at OD_*i*_ = 1:1, Met–Trp4^A–B:*FLO1*^:Mctrl, *P* = 0.0017. In **e**, at OD_*i*_ = 10:1, Lys–Ade^B–A^:Nctrl^B–A^, *P* < 0.0001; Nctrl^B–A:*FLO1*^:Nctrl^B–A^, *P* = 0.0081; Lys–Ade^B–A^:Mcrtl, *P* = 0.0127; at OD_*i*_ = 1:1, Nctrl^A^^−B:*FLO1*^:Nctrl^A–B^, *P* < 0.0001; Met–Trp4^A–B:*FLO1*^:Met–Trp4^A–B^, *P* < 0.0001; Met–Trp4^A–B:*FLO1*^:Mctrl, *P* < 0.0001; Met–Trp4^A–B^:Mctrl *P* = 0.0086. In **f**, at OD_*i*_ = 1:1, Met–Trp4^A–B:*FLO1*^:Met–Trp4^A–B^, *P* < 0.0001; Met–Trp4^A–B:*FLO1*^:Mctrl, *P* < 0.0001. Source data

To demonstrate the practical application of our toolkit in a metabolic engineering context, we chose production of the high-value food antioxidant resveratrol as a proof of concept^41,42^. The resveratrol synthesis pathway involves three key enzymes: FjTAL, At4CL and VvVST1^43^, which we divided into two modules that we proved were able to improve production in synthetic cross-feeding cocultures^4^. We therefore generated strains combining the production modules (division of labor), auxotrophies enabling cross-feeding and cell–cell adhesion systems, and hypothesized that, in some cases, metabolic fluxes could be optimized and resveratrol production could be improved (Fig. 5a). Module A contained FjTAL, catalyzing L-tyrosine to *p*-coumaric acid, while module B contained At4CL and VvVST1, converting *p*-coumaric acid to resveratrol. In each coculture, one strain was engineered with module A (*FjTAL*) and the other with module B (*At4CL* and *VvVST1*) (Fig. 5a). To investigate the impact of cross-feeding and cell adhesion on resveratrol production, we used three coculture pairs (Lys–Ade, Trp2–Trp4 and Met–Trp4), each with or without the incorporating of FLO1_co_ adhesion pairs (Fig. 5b). Initial OD ratios of 10:1 for Lys–Ade, and 1:1 for Trp2–Trp4 and Met–Trp4 were chosen based on the previously observed growth dynamics of each pair. Biomass, resveratrol and *p*-coumaric acid levels were quantified at the end of the fermentation. We included noncross-feeding control pairs (Nctrl^A–B^, Ncrtl^B–A^, Ncrtl^A–B:*FLO1*^ and Ncrtl^B–A:*FLO1*^) and a monoculture control (Mctrl) expressing the entire resveratrol pathway for comparison. The superscript ‘A–B’ denotes module A in cell 1 and module B in cell 2, while ‘B–A’ indicates module B in cell 1 and module A in cell 2.

Interestingly, when looking at biomass formation, we found that despite having seen that *FLO1* expression increased growth in the cross-feeding cocultures (Extended Data Fig. 3d), the coexpression of *FLO1* and the resveratrol genes led to a growth reduction in half of the tested cross-feeding cocultures (especially in the pair Lys–Ade), and in some of the coculture controls with no cross-feeding, which could originate from the metabolic burden (Fig. 5c). Unlike the spontaneously established syntrophic yeast communities formed by the Trp2–Trp4 and Met–Trp4 pairs^12^, the Lys–Ade pair requires overexpression of both *ade4op* and *lys21op* to maintain cross-feeding^4^, thereby imposing a burden on cell fitness.

When looking at production, we also observed that in the coculture control Ncrtl^B–A:*FLO1*^ at a 10:1 ratio and Ncrtl^A–B:*FLO1*^ at a 1:1 ratio, the presence of *FLO1* led to a 5.39- and 3.55-fold increase in resveratrol compared to Ncrtl^B–A^ at a 10:1 ratio (*P* = 0.0081) and Ncrtl^A–B^ at a 1:1 ratio (*P* < 0.0001), respectively (Fig. 5d,e), suggesting a benefit of cell–cell adhesion on the division of labor in the absence of cross-feeding. This was not the case in the remaining control pair Ncrtl^A–B^ at a 10:1 ratio, where no appreciable changes were observed. This suggests that both initial ratio and cell adhesion contribute to bioproduction dynamics in cocultures with division of labor.

Of the Lys–Ade cocultures, Lys–Ade^B–A^, where module B was expressed in cell 1, was the best performing and produced significantly more resveratrol (22.5% increase, *P* = 0.0127) than Mctrl (Fig. 5e). However, the coculture Lys–Ade^B–A:*FLO1*^, with the same pathway distribution but also expressing *FLO1*, produced only a small amount of detectable resveratrol, with a higher accumulation of the intermediate *p*-coumaric acid (Fig. 5d), suggesting that the division of labor is not well balanced. Similarly, the Trp2–Trp4 cocultures showed increased *p*-coumaric acid accumulation (especially when *FLO1* was expressed) and limited resveratrol production.

Notably, the pair Met–Trp4, in the presence of *FLO1*, always increased production. In particular, coculture Met–Trp4^A–B:*FLO1*^ produced 0.67 μM of resveratrol, a 2.47-fold increase compared to Mctrl (*P* < 0.0001), an 8.68-fold increase compared to Ncrtl^A–B^ at a 1:1 ratio (*P* < 0.0001), a 2.45-fold increase compared to Ncrtl^A–B:*FLO1*^ at a 1:1 ratio (*P* < 0.0001) and a 1.64-fold increase compared to Met–Trp4^A–B^ (*P* < 0.0001) (Fig. 5d). Moreover, comparing resveratrol bioproduction per cell (normalizing by OD_600__nm_), the Met–Trp4^A–B:*FLO1*^ coculture achieved a value that was 4.32 times higher than Mctrl (*P* < 0.0001) and 2.58 times higher than the Met–Trp4^A–B^ coculture (*P* < 0.0001) (Fig. 5f). These results suggest that resveratrol production can be notably improved in some cross-feeding cocultures, particularly in Met–Trp4 with *FLO1* expression. These results demonstrate the potential synergies between cross-feeding, cell adhesion and division of labor in improving bioproduction in synthetic yeast communities.

It is important to note that while certain conditions, such as the Lys–Ade^B–A^ and Met–Trp4^A–B:*FLO1*^ cocultures, resulted in higher final resveratrol titers compared to Mctrl, they also led to increased accumulation of the intermediate *p*-coumarate. From an industrial perspective, this reflects a limitation in precursor conversion efficiency, which could adversely affect overall process economics. Although this study primarily serves as a proof of concept, these results indicate that future optimization can increase overall yields.

Moreover, under some conditions (Fig. 5c), such as the Met–Trp4^A–B:*FLO1*^ coculture, substantial resveratrol production was achieved despite showing lower OD_600nm_ values. This observation suggests that a greater proportion of the feedstock was directed toward product synthesis rather than biomass accumulation, a characteristic that may be beneficial for industrial applications.

## Discussion

This study notably advances synthetic biology by developing a series of building blocks and strains for engineered cell–cell adhesion and coculture in yeast. This toolbox includes a base yeast strain (007Δ) providing a clean background for synthetic adhesion systems, three pairs of cross-feeding cocultures and 16 adhesion pairs, with potential for further expansion. The combination of adhesion and cross-feeding enable more precise manipulation of multicellular assemblies. A higher understanding of multicellular assembly has broad implications for studying the development of biofilms, higher organisms and microbial communities^8,15,44,45^.

Using this toolkit, we created and characterized the growth dynamics of synthetic yeast cocultures across three adhesion pair systems, showcasing the role of specific adhesion systems, such as flocculation, agglutination and surface display in facilitating the formation of multicellular aggregates to enhance cross-feeding efficiency. This provides an additional way to study the intricate balance between genetic control and physical interactions in microbial consortia. Systematic investigation of aggregate morphology and its impact on metabolic exchange represents an important direction for future research. Through quantitative analysis of adhesion specificity, we revealed the crucial role of both nonspecific and specific adhesion molecules in enhancing intercellular adhesion. The ability to engineer complex multicellular structures and patterns with precision has potential applications in tissue engineering, metabolic pathway optimization and the design of artificial organs^13,39,45,46^.

Our results identified the widely studied flocculation gene *FLO1* (refs. ^30,32^) as a key factor in engineering cell adhesion and potentially facilitating nutrient exchange in synthetic yeast cocultures, thereby improving the growth or production of natural products. In our previous study, we investigated three synthetic yeast cocultures (Ade–Lys, Ade–Trp and Trp–Lys) and showed that combining cross-feeding with division of labor increased the resveratrol titer by up to 2.16-fold (0.79 μM) compared to the monoculture control Mctrl (0.25 μM)^4^. Building on this, the current study revisited Ade–Lys and introduced two new coculture pairs (Trp2–Trp4 and Met–Trp4), integrating cross-feeding, division of labor and an adhesion strategy. This approach further improved resveratrol titer, achieving up to a 2.47-fold increase over Mctrl.

Although the absolute titer, yield and productivity achieved in this study are moderate, the approach we propose, combining division of labor, cross-feeding and cell–cell adhesion, is broadly generalizable to metabolic engineering applications. Division of labor is particularly advantageous for complex, high-burden or toxic intermediate pathways, and has already enhanced the production of compounds such as resveratrol, raspberry ketone and alkaloids across *E. coli* and yeast coculture systems^4,9,41,42,47–49^. Our framework provides modular design strategies, including pathway partitioning, growth balancing, metabolite transfer optimization and spatial organization via adhesion. These principles can be adapted to improve titers and yields for other high-value compounds.

Division of labor and cross-feeding strategies are particularly effective for alleviating metabolic burden and enhancing biosynthetic efficiency^9,41,42,47^. However, these approaches require careful balancing of growth rates and metabolite exchange to maintain stability. Other tools such as quorum sensing-based communication circuits enable autonomous coordination of strain behaviors^50^, but face challenges such as cross-talk and genetic burden. Recent advances in optogenetic and cybergenetic control systems allow dynamic^51^, real-time adjustment of population ratios, offering high precision but at the cost of technical complexity and scalability. These developments also contribute to the broader field of microbiome engineering^52^, where synthetic communities are designed to perform coordinated functions in complex environments. Overall, our modular coculture approach offers broad generalizability and ease of implementation for metabolic engineering and can be further strengthened by integrating dynamic regulation and spatial control strategies.

Furthermore, our study demonstrates the ability to control population dynamics in synthetic cocultures through the manipulation of initial cell ratios in combination with cell–cell adhesion systems. This approach is important for achieving targeted cellular outcomes and elucidating complex metabolic interactions, particularly in engineered microbial communities.

In addition, our experiments revealed key insights into yeast coculture dynamics. Certain combinations of cross-feeding and cell–cell adhesion, particularly those involving the *FLO1* gene, led to synergistic effects, enhancing cell growth. The further combination with division of labor for resveratrol production showed that yeast coculture pairs like Met–Trp4 increased resveratrol production. However, this effect was not universal, underscoring the importance of careful strain selection and environmental optimization for desired metabolic outputs. These findings highlight the potential of spatial control via engineered cell–cell adhesion in enhancing metabolic efficiency and production in microbial communities. Overall, our results demonstrate that combining efficient cross-feeding yeast cocultures with engineered cell–cell adhesion, coupled with division of labor, can synergistically improve the production of the high-value aromatic food antioxidant resveratrol, emphasizing the power of integrated strategies in optimizing the performance of microbial communities. Besides, there are many factors involved in the phenotype that affect bioproduction, making accurate predictions and mechanistic understanding challenging. These factors include the rate of metabolite exchange during cross-feeding, differences in growth rates, inoculation ratios, the metabolic burden of pathway expression, expression of adhesion genes and the overexpression of *ade4op* and *lys21op*, among others.

In conclusion, our research enriches the field of synthetic biology by providing a framework for examining and manipulating cell–cell adhesion and synthetic syntrophic cocultures in yeast. Future investigations can explore the implications of these findings, particularly in biomanufacturing and biomedical applications, but also in further understanding microbial communities and ecology. In particular, this work paves the way to future innovations and discoveries in the field of tissue and metabolic engineering.

## Methods

### Strains, media and chemicals

*Escherichia coli* Turbo Comp (NEB) was used for routine bacterial cloning and plasmid propagation. For *E. coli* selection and growth, we used LB medium, maintained at 37 °C and supplemented with appropriate antibiotics: ampicillin at 100 μg ml^−1^, chloramphenicol at 34 μg ml^−1^ or kanamycin at 50 μg ml^−1^ (ref. ^24^). The wild-type (WT) yeast strain utilized in our study was a derivative of BY4741 (MATa *his3*Δ1 *leu2*Δ0 *met15*Δ0 *ura3*Δ0). We cultured yeast cells in three media: yeast extract peptone dextrose (YPD) medium, synthetic complete dextrose (SD) medium and SM medium. The YPD medium composition included 10 g l^−1^ yeast extract, 20 g l^−1^ peptone and 20 g l^−1^ glucose. The SD medium contained 6.7 g l^−1^ yeast nitrogen base without amino acids, 1.4 g l^−1^ yeast synthetic dropout medium supplement without histidine, leucine, tryptophan and uracil (Sigma-Aldrich) and 20 g l^−1^ glucose. When required, media were supplemented with histidine (20 mg l^−1^), leucine (120 mg l^−1^), tryptophan (20 mg l^−1^) and uracil (20 mg l^−1^). SM medium was prepared by combining 6.7 g l^−1^ yeast nitrogen base without amino acids, 20 g l^−1^ glucose and amino acids according to the recipes described by Mülleder et al.^53^. To create solid plates, bacteriological agar (2%) was added to YPD, SD or SM media. For preservation, yeast strains and *E. coli* harboring plasmids were stored at −80 °C in 25% (v/v) glycerol. All reagents, chemicals and analytical standards, including resveratrol and p-coumaric acid, used in this study, are detailed in Supplementary Table 3.

### Plasmid construction and bacterial transformation

In this study, all plasmids were constructed using the MoClo-Yeast Toolkit system, following the methodology outlined by Lee et al.^54^. Unless stated otherwise, all part sequences were either polymerase chain reaction (PCR) amplified or synthesized with the exclusion of internal BsmBI, BsaI, BpiI and NotI recognition sequences. For the construction of all plasmids referenced in Supplementary Table 4, a Golden Gate gene assembly approach was used. Before initiating the experiments, all component parts were standardized to equimolar concentrations of 50 fmol ml^−1^ (50 nM). The Golden Gate reaction mixtures were carefully prepared, comprising 0.1 μl of the backbone vector, 0.5 μl of each plasmid, 1 μl of T4 DNA ligase buffer (Promega), 0.5 μl of T7 DNA Ligase (NEB), 0.5 μl of the appropriate restriction enzyme (either BsaI or BsmBI from NEB). The total volume was brought to 10 μl with the addition of nuclease-free water. The reaction mixtures were subjected to incubation in a thermocycler under the following condition: a 2-min cycle at 42 °C followed by a 5-min cycle at 16 °C, this sequence repeated for a total of 25 cycles. Subsequently, a final digestion step was performed at 60 °C for 10 min, followed by heat inactivation at 80 °C for 10 min. Post-reaction, the mixtures were then used for *E. coli* transformation using a TSS (Transformation storage solution) protocol for KCM (KCl, CaCl_2_, MgCl_2_) chemical transformation^55^. Finally, the transformed *E. coli* cells were plated on LB agar plates, supplemented with the relevant antibiotics for selection.

### Yeast transformation and colony PCR validation

Yeast transformation was performed using a standard lithium acetate (LiOAc) protocol^56^. Chemically competent yeast cells were prepared as follows. Freshly isolated colonies were cultured in YPD medium at 30 °C and 250 rpm overnight to reach saturation. The following morning, the cells were diluted 1:100 in 10 ml of fresh YPD in a 50-ml conical tube and incubated for 4–6 h until reaching an OD_600nm_ of 0.8–1.0. The cells were then pelleted, washed once with an equal volume of 0.1 M LiOAc and resuspended in 600 μl of 0.1 M LiOAc. Aliquots of 100 μl of cells were transferred into individual 1.5-ml tubes, pelleted and prepared for yeast transformation.

For the transformation, the cells were resuspended in 64 μl of a DNA–salmon sperm DNA mixture containing 10 μl of boiled salmon sperm DNA (Invitrogen), NotI-digested plasmids and ddH_2_O. This mixture was then combined with 294 μl of a PEG–LiOAc mixture consisting of 260 μl of 50% (w/v) PEG-3350 and 36 μl of 1 M LiOAc. The yeast transformation mixture was heat-shocked at 42 °C for 40 min, pelleted, resuspended in 200 μl of 5 mM CaCl_2_, and incubated for 10 min before plating onto selection plates. Yeast colonies should appear after incubation at 30 °C for 2–3 days (or longer for complex transformations or large genes).

The success of yeast transformation was verified by colony PCR using the Phire Plant Direct PCR Master Mix (F160L, Thermo Fisher). For each yeast transformation, between three and five isolated colonies were selected and resuspended in 20–50 μl of sterile water in PCR tubes. Each 10-μl PCR reaction system included 1 μl of the cell suspension, 5 μl of 2× Phire Plant Direct PCR Master Mix, 0.5 μl of the forward primer, 0.5 μl of the reverse primer and 3 μl of ddH_2_O. The PCR reactions were performed using the ProFlex PCR System (Thermo Fisher) under the recommended conditions for Phire Plant polymerase: initial denaturation at 95 °C for 5 min, followed by 35 cycles of denaturation at 98 °C for 5 s, annealing at *X* °C for 5 s (*X* represents the optimum annealing temperature for each primer pair), extension at 72 °C for 20 s per kilobase, and a final extension at 72 °C for 1 min. The 10-μl PCR reaction was then verified by agarose gel electrophoresis.

### Genome editing of yeast using CRISPR–Cas9

Auxotrophic yeast strains were constructed using iterative markerless CRISPR–Cas9 genome engineering, following the method described by Shaw et al.^24,57^. We used a sequential gene knockout approach to systematically delete seven specific genes, *FLO1*, *FLO5*, *FLO10*, *FLO11*, *AGA1*, *AGA2* and *SAG1*, in the BY4741 yeast strain. Detailed information on the guide RNAs and landing pads can be found in the primer list provided in Supplementary Table 5.

A two-plasmid system was used for genome editing, following the protocol described by Shaw et al.^24^. This system consisted of a gRNA expression plasmid (pWS2069), using the MoClo-Yeast Toolkit system^54^, and three CRISPR–Cas9 expression plasmids (pWS158, pWS171, pWS172), each containing different selectable marker (Ura3, Leu2, His3) under the control of the PGK1 promoter and terminator. Plasmid transformation was achieved by digesting the CRISPR–Cas9 and gRNA expression plasmids with BsmBI or BbsI enzymes, respectively.

All gRNAs were designed using Benchling’s CRISPR Design Tool, with the full list provided in Supplementary Table 5. The 20-bp target sequences were designed to exclude internal BsaI, BsmBI or NotI restriction sites to facilitate downstream cloning and transformation. gRNA assemblies were set up in 10-µl reactions containing 1 μl of primer (100 μM), 1 μl of 10× T4 DNA ligase buffer (Promega), 0.5 μl of T4 PNK (NEB) and 7.5 μl of H_2_O. The mixture was incubated for 1 h at 37 °C, then combined with the complementary oligonucleotide reaction (the sense and antisense oligonucleotide reactions, each 10 μl) and diluted to 200 μl with H_2_O. The oligonucleotides were annealed using a slow temperature ramp-down protocol: 96 °C for 6 min, followed by a ramp down of 0.1 °C s^−1^ to 20 °C and a 20 °C hold. The resulting product was ligated into the pWS2069 vector using a Golden Gate assembly reaction. Donor DNA for multiplexed editing was generated by cloning the target gene sequence into the pYTK001 part entry vector, which provides 500 bp of homology flanking the insert.

### Monoculture and coculture preparation for microplate reader assays and bioproduction

#### Preparation of seed culture and OD adjustment for coculture

Freshly isolated colonies of wild-type or verified engineered yeast strains were precultured overnight in 2 ml of selective SC media at 30 °C and 250 rpm. The following day, 1 ml of saturated preculture was pelleted in a 1.5-ml tube, centrifuging at 3,000*g* for 1 min. The cell pellet was then washed three times with SM medium, centrifuging at 3,000*g* for 1 min each time, and finally resuspended in 1 ml of SM medium. A 100-μl aliquot was then diluted tenfold, and the OD_600nm_ was measured using cuvettes and a UV–visible spectrophotometer (Biochrom WPA Lightwave II, Biochrom Ltd). The remaining 900 μl of cells were pelleted and resuspended in SM medium to an equivalent OD_600nm_ of 20, then used for monoculture and coculture setups as described below.

#### Monoculture and coculture preparation for microplate reader assay

For monoculture experiments, 3 μl of washed cells (OD_600nm_ = 20) were inoculated into 147 μl of SM medium in a 96-well plate (cat. no. 655090, Greiner Bio-One). Specific auxotrophic amino acids were added as positive controls for monoculture, while negative controls received no amino acids supplementation. The resulting cultures had an initial OD_600nm_ of 0.4 (measured by spectrometry) in 150 μl. Cocultures were prepared by inoculating 3 μl of each washed strain (OD_600nm_ = 20) into 144 μl of SM medium, resulting in a total OD_600nm_ of 0.8 per 150 μl culture. In two-member cocultures with varying initial ratios, we adjusted the cell density of each member to achieve 10:1, 1:1 and 1:10 ratios.

#### Resveratrol production in monoculture and two-member coculture

For bioproduction experiments, monocultures and cocultures were performed in 500-μl volumes in 96-well deep plates. Monocultures were set with an OD_600nm_ of 0.4 by inoculating 10 μl of washed seed culture (OD_600nm_ = 20) into 490 μl of SM medium. Cocultures were set with a combined OD_600nm_ of 0.8 (0.4 per strain) by inoculating 20 μl of mixed washed seed cultures (OD_600nm_ = 20) into 480 μl of SM medium. Initial strain ratios of 1:1, 1:10 and 10:1 cocultures were tested. All cultures were incubated at 30 °C and 250 rpm for 48 h in InforsHT Multitron incubators.

### OD measurement and microplate reader assay

The OD_600nm_ values of overnight seed cultures in 14-ml tubes were measured using cuvettes in a UV–visible spectrophotometer (Biochrom WPA Lightwave II) after diluting the samples 10–20 times. To avoid interference from simultaneous fluorescence measurements, the OD_700nm_ values of cultures grown in (deep) 96-well plates were measured using a SPARK multimode microplate reader (Tecan) controlled by Magellan Standard software. All samples were thoroughly vortexed or mixed using a pipette before OD measurement. For real-time monitoring, all cultures were incubated at 30 °C with double orbital continuous shaking at 270 rpm in SPARK multimode microplate reader (Tecan). Measurements were taken every 30 min with the following settings: OD_700nm_, absorbance at 700 nm; sfGFP (excitation at 485 nm, emission at 535 nm); and mRuby2 (excitation at 560 nm, emission at 620 nm). Estimated OD_700nm_ of the sfGFP-tagged population (GOD) and estimated OD_700nm_ of the mRuby2-tagged population (ROD) were calculated using the standard curves between OD_700nm_ values and fluorescence (sfGFP and mRuby2) intensities (Supplementary Table 2)^4^. Unless explicitly stated otherwise, all OD_600nm_ values were obtained from spectrophotometer readings, whereas all OD_700nm_ values were measured using the Tecan microplate reader.

### Yeast flocculation assay

The yeast strains used in this study are listed in Supplementary Table 6. To assess flocculation and adhesive growth, nonadhesive BY4741 mutants (*flo1*Δ, *flo5*Δ, *flo10*Δ, *flo11*Δ, *aga1*Δ, *aga2*Δ and *sag1*Δ) were transformed with appropriate adhesion gene constructs. Adhesion assays on agar and plastic surfaces were conducted following previously described methods^58,59^. Briefly, 3 ml of yeast cultures of cell 1 and cell 2 (OD_600nm_ = 10) were prepared in SD medium, mixed in a test tube, and allowed to settle for 4 h. The OD_600nm_ was then measured from a 200-μl sample taken at the midpoint of the liquid height.

### Fluorescence microscopy and time-lapse imaging

Confocal microscopy was conducted using a Nikon Eclipse Ti microscope with an S Plan Fluor ELWD 20× DIC N1 objective. Images were acquired and analyzed with NIS-Elements AR (v.5). Excitation and emission wavelengths were optimized for sfGFP (535 nm), mTagBFP2 (405 nm) and mRuby2 (610 nm) with manual adjustments to minimize bleed-through. Before imaging, 600–1,200-μl cell mixtures were allowed to settle in 1.5-ml microcentrifuge tubes for ~1 h at room temperature. Subsequently, around 10 μl of settled aggregates were carefully transferred to a microscope slide chamber using a wide-orifice pipette tip, sealed with a coverslip and Thomas Lubriseal stopcock grease. To ensure accurate three-color images, bleed-through from the blue to green channels was corrected by subtracting the blue channel signal from the green channel signal.

For time-lapse imaging of yeast cell–cell adhesion and coculture, cell 1 and cell 2 cultures were adjusted to an initial OD_600nm_ of 0.2 in SM medium. Then, 200 µl of each culture were combined in a 1.5-ml centrifuge tube and allowed to mix for 1 h. A 20-µl aliquot of the mixed culture was gently placed onto a 35-mm µ-Dish, covered with a coverslip and sealed with 1% low-melting-point agarose. Time-lapse imaging was performed using a Nikon Eclipse Ti microscope with an S Plan Fluor ELWD 20× DIC N1 objective. Images were acquired at 30-min intervals over a 36-h incubation period at 30 °C (ref. ^60^).

### Quantification of microscopic cell cluster types and coculture growth

For the two-cell mixed system, adhesion patterns were classified into five distinct types based on aggregation forms: multicellular with red and green, multicellular with red or green, tricellular–bicellular with both red and green, tricellular–bicellular with red or green and single cell. For the three-cell mixed system, subsequent analysis of cell composition identified four categories: tricolor cell cluster (containing all three cell types), bicolor with blue cluster (cell 3 combined with either cell 1 or cell 2), cluster with red and green cluster (cell 1 and cell 2 only), and monochrome cells (a single cell type). Quantification is based on the proportion of each cell type relative to the total number of cells, using six confocal images obtained from slices of three biologically independent replicates. The Fiji software package was used to process and analyze the images.

In the time-lapse quantification of cell density, only the fold increase in cell density at 24 h relative to the initial density was analyzed, because of the notable expansion of cell growth at later stages. The *FLO1*-introduced sample, characterized by red and green cells forming clusters, was selected as the representative image. Quantification results were obtained by averaging measurements from confocal slices across three replicates.

### Visualization of 2D patterns in synthetic yeast cocultures

Yeast cultures were carefully seeded in desired shapes using a pipette tip and incubated at 30 °C for 4 days. To create specific patterns, cell 1 (007Δ *FLO1 sfGFP*) was pipetted onto SD agar in the desired pattern. A liquid culture of cell 2 (007Δ *FLO1 mRuby2*) (OD_600nm_ = 2.0) was then added to the plate and incubated for 2 h without disturbance. The cell 2 solution was then discarded, SD medium was added and the plate was gently washed. To create cross-feeding patterns, 200 µl of cell 2 (*ade8*Δ (or *trp4*Δ) *FLO1 mRuby2*) was spread on SM agar, and cell 1 (*lys2*Δ (*trp2*Δ or *met14*Δ) *FLO1 sfGFP*) was seeded in the desired shape and incubated at 30 °C for 4 days. Yeast cell patterns were imaged using an Amersham Typhoon scanner at 10 µm resolution with a far-blue light gel transilluminator with amber filter, and data collected through Scanner Control software v.3.0^39^.

### Liquid chromatography–mass spectrometry analysis of metabolites in the resveratrol synthesis pathway

A 300-μl cell culture was mixed with an equal volume of ethanol and incubated at 700 rpm and 30 °C for 5 min. Subsequently, the mixture was centrifuged at 2,500*g* for 30 min to obtain the supernatants. These supernatants were then loaded into a 96-well sample plate for LC–MS analysis, following the established method described previously^9,61^. The LC–MS analysis was conducted using an Agilent 1290 Infinity system equipped with an online diode array detector and an Agilent 6500 quadruple time-of-flight mass spectrometer. A temperature of 25 °C was maintained throughout the analysis. An Agilent Eclipse Plus C18 column (2.1 × 50 mm, 1.8 μm particle size) was used with a solvent flow rate of 0.2 ml min^−1^. The LC gradient consisted of buffer A (0.1% formic acid) and buffer B (0.1% formic acid in acetonitrile). The gradient started at 2% buffer B and increased to 98% over 2.5 min, followed by a 1-min hold at 98% buffer B. For the injection, 1 μl of the prepared samples was used. Spectra were recorded between a mass range of 90–1,000 *m*/*z* at a rate of three spectra per second. Quantitation of metabolites was performed by comparing the MS peak area of precursor or fragment ions to the prepared calibration curves of standards, including *p*-coumaric acid and resveratrol. Resveratrol samples were detected in negative ion mode. Agilent MassHunt v.10 was used to collect LC–MS data. MassHunter Quantitative software v.10 was used to analyze LC–MS data. To ensure the reliability of the results, the experiment was repeated three times using independent biological samples. The error bars represent the standard deviation calculated from these replicates.

### Statistical analysis and reproducibility

Unless specifically stated otherwise, all data analyses were performed using Microsoft Excel 365 and Prism 9.5.0 (GraphPad) software. The error bars in the figures correspond to the standard deviation from *n* = 3 biologically independent samples as described in figure legend. Statistical tests were conducted using one-way ANOVA, followed by Tukey’s multiple comparisons test with 95% confidence intervals and *P* values are noted.

### Reporting summary

Further information on research design is available in the Nature Portfolio Reporting Summary linked to this article.

## Online content

Any methods, additional references, Nature Portfolio reporting summaries, source data, extended data, supplementary information, acknowledgements, peer review information; details of author contributions and competing interests; and statements of data and code availability are available at 10.1038/s41589-025-02081-1.

## Supplementary information

Supplementary Information Supplementary Figs. 1–13.

Reporting Summary

Supplementary Tables 1–6 Supplementary tables, including metabolite supplementation for cell growth assays with stock-solution preparation notes; standard curves converting sfGFP and mRuby2 fluorescence to OD and comprehensive lists of reagents and chemicals, plasmids, oligos and primers, and strains used in this study.

Supplementary Video 1 Time-lapse videos of the *lys2*Δ-*ade8*Δ coculture over 48 hours. Green cells: 007Δ *lys2*Δ *sfGFP*; Red cells: 007Δ *ade8*Δ *mRuby2*.

Supplementary Video 2 Time-lapse videos of the *lys2*Δ-*ade8*Δ with *FLO1* coculture over 48 hours. Green cells: 007Δ *lys2*Δ *FLO1 sfGFP*; Red cells: 007Δ *ade8*Δ *FLO1 mRuby2*.

Supplementary Video 3 Time-lapse videos of the *trp2*Δ-*trp4*Δ coculture over 36 hours. Green cells: 007Δ *trp2*Δ *sfGFP*; Red cells: 007Δ *trp4*Δ *mRuby2*.

Supplementary Video 4 Time-lapse videos of the *trp2*Δ-*trp4*Δ with *FLO1* coculture over 36 hours. Green cells: 007Δ *trp2*Δ *FLO1 sfGFP*; Red cells: 007Δ *trp4*Δ *FLO1 mRuby2*.

Supplementary Video 5 Time-lapse videos of the *met14*Δ-*trp4*Δ coculture over 36 hours. Green cells: 007Δ *met14*Δ *sfGFP*; Red cells: 007Δ *trp4*Δ *mRuby2*.

Supplementary Video 6 Time-lapse videos of the *met14*Δ-*trp4*Δ with *FLO1* coculture over 36 hours. Green cells: 007Δ *met14*Δ *FLO1 sfGFP*; Red cells: 007Δ *trp4*Δ *FLO1 mRuby2*.

Supplementary Video 7 Time-lapse videos of the 007Δ coculture over 24 hours. Green cells: 007Δ *sfGFP*; Red cells: 007Δ *mRuby2*.

Supplementary Video 8 Time-lapse videos of the 007Δ with *FLO1* coculture over 24 hours. Green cells: 007Δ *FLO1 sfGFP*; Red cells: 007Δ *FLO1 mRuby2*.

Supplementary Data 1 Statistical source data for Supplementary Figs. 3d, 7b and 8–13.

## Source data

Source Data Fig. 1 Statistical source data.

Source Data Fig. 2 Statistical source data.

Source Data Fig. 4 Statistical source data.

Source Data Fig. 5 Statistical source data.

Source Data Extended Data Fig. 3 Statistical source data.

## Data Availability

All data generated or analyzed during this study are included in the published article and its Supplementary Information. Data are available from the corresponding author upon request. Source data are provided with this paper.
