# Supplementary material for: Programmable cell–cell adhesion in synthetic yeast communities for improved bioproduction
Source: Nat Chem Biol. 2026 Jan 5;22(7):1057–66. doi: 10.1038/s41589-025-02081-1 (PMC13303087; doi:10.1038/s41589-025-02081-1)
Supplement: Supplementary file 1 — Supplementary Figs. 1–13. [file 41589_2025_2081_MOESM1_ESM.pdf]

nature chemical biology

Supplementary information https://doi.org/10.1038/s41589-025-02081-1

Programmable cell–cell adhesion in synthetic yeast communities for improved bioproduction

In the format provided by the authors and unedited

Supplementary information

Contents

Supplementary Fig. 1 Assembly architecture of surface display vector.. 2

Supplementary Fig. 2 Schematic illustration of yeast surface display supplemented with *FLO1* gene introduction. 3

Supplementary Fig. 3 Schematic representation and confocal microscopy images of multicellular patterning outcomes in engineered yeast strains... 4

Supplementary Fig. 4 Two engineered cells (Cell 1 and Cell 2) were used for assessing their ability to form 2D patterns. 6

Supplementary Fig. 5 Colony growth assays reveal cross-feeding interactions and auxotrophic requirements in synthetic yeast co-cultures.. 7

Supplementary Fig. 6 Cell patterning test. 8

Supplementary Fig. 7 Time-lapse microscopy reveals dynamic cell growth and patterning in synthetic yeast co-cultures with engineered adhesion.. 9

Supplementary Fig. 8 Impact of cross-feeding pairs and inoculation ratios on the growth dynamics of Lys-Ade co-cultures with engineered adhesion. 10

Supplementary Fig. 9 Impact of intercellular adhesion pairs on the Lys-Ade co-culture system under dynamic condition. 11

Supplementary Fig. 10 Impact of cross-feeding pairs and inoculation ratios on the growth dynamics of Trp2-Trp4 co-cultures with engineered adhesion... 12

Supplementary Fig. 11 Impact of intercellular adhesion pairs on the Trp2-Trp4 co-culture system under dynamic condition. 13

Supplementary Fig. 12 Impact of cross-feeding pairs and inoculation ratios on the growth dynamics of Met-Trp4 co-cultures with engineered adhesion.. 14

Supplementary Fig. 13 Impact of intercellular adhesion pairs on the Met-Trp4 co-culture system under dynamic condition. 15

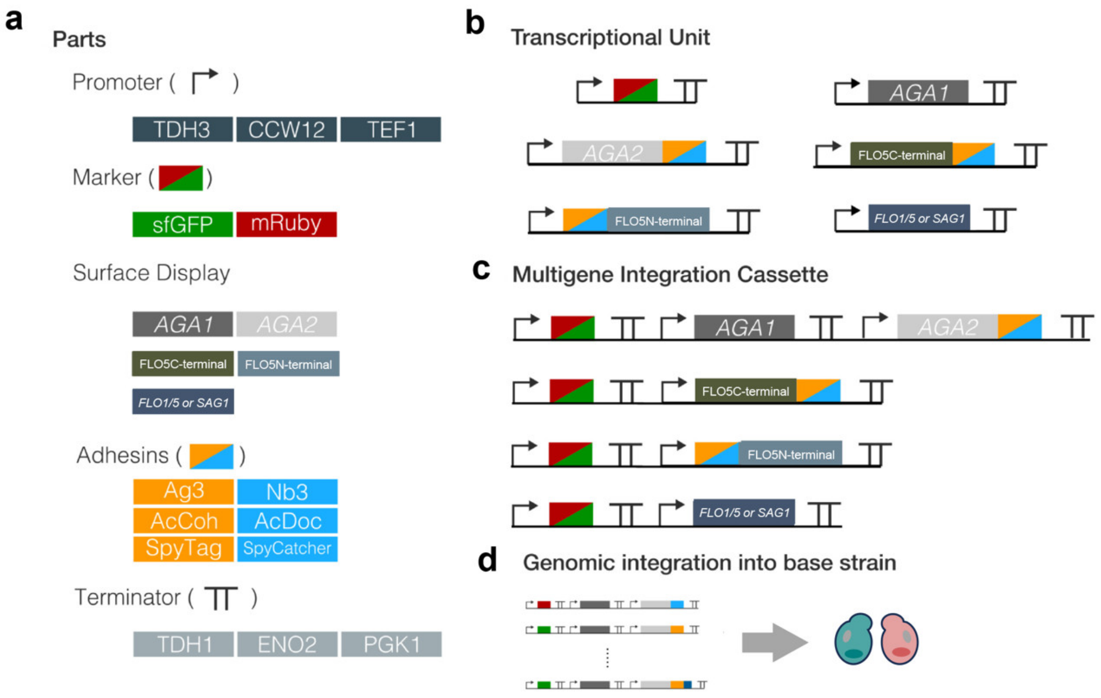

Supplementary Fig. 1 Assembly architecture of surface display vector.

a, Library of promoter, maker, surface display-ORF, adhesion tags, and terminator parts for surface display systems. **b,** Transcriptional unit combinations used in surface display systems. **c,** Multigene integration cassette array for surface display systems. **d,** Genomic integration into base strains.

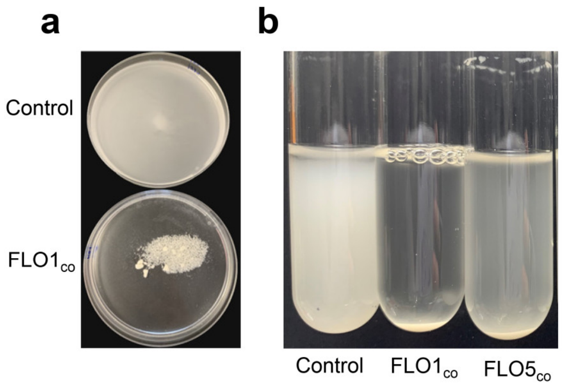

Supplementary Fig. 2 Schematic illustration of yeast surface display supplemented with *FLO1* gene introduction.

a, High-density cell flocculation phenotype. To visualize enhanced flocculation, equal volumes of Cell 1 and Cell 2 cultures (OD_{600nm} = 20) in Synthetic Minimal (SM) medium were combined on a culture plate and incubated at 30°C with shaking (60 rpm) for 1 hour. Yeast overexpressing the gene *FLO1* exhibited substantial flocculation, resulting in a clear supernatant. **b,** Schematic diagram illustrating yeast flocculation phenotypes during fermentation. 200 µL Cell 1 and Cell 2 (OD_{700nm} = 20) were cultured in 4 mL SM medium at 30°C with shaking (260 rpm) for 14 hours. Overexpression of *FLO1* and *FLO5* induced cell flocculation and precipitation, with *FLO1* exhibiting a stronger effect.

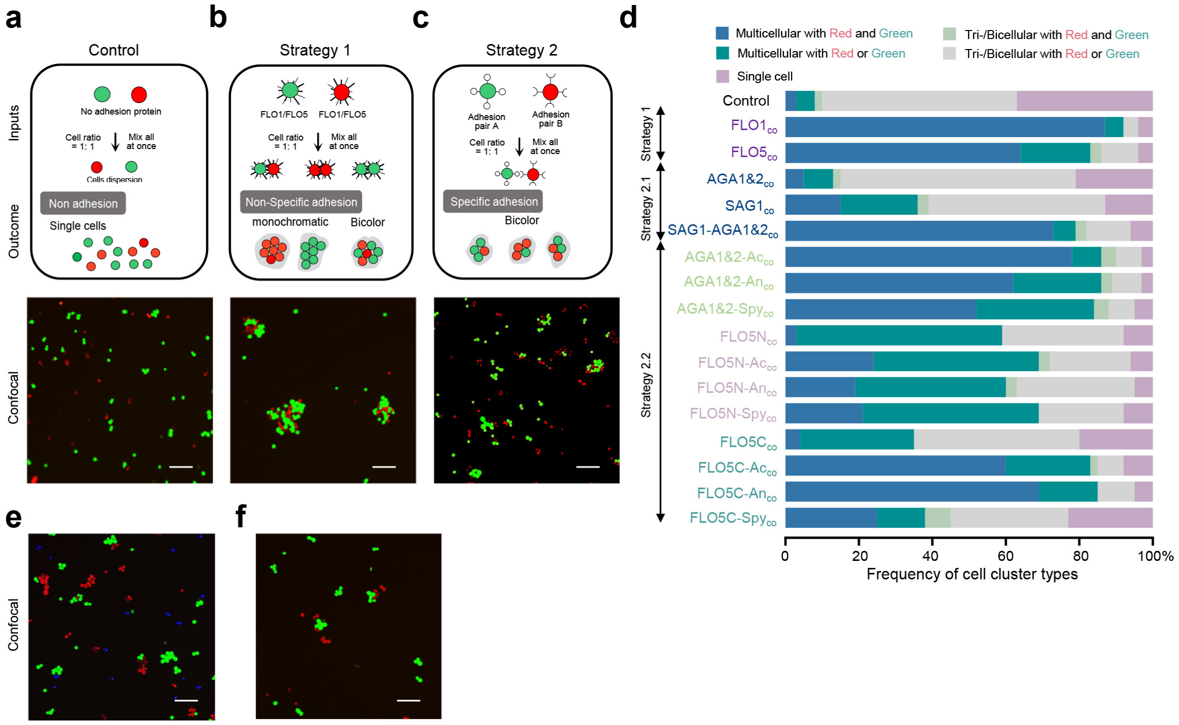

Supplementary Fig. 3 Schematic representation and confocal microscopy images of multicellular patterning outcomes in engineered yeast strains.

a, Deletion of seven adhesion-related genes (*FLO1*, *FLO5*, *FLO10*, *FLO11*, *AGA1*, *AGA2*, *SAG1*) leads to loss of adhesion in Cell 1 (007Δ *sfGFP*, Green) and Cell 2 (*007Δ mRuby2,* Red) and a dispersed cell phenotype (scale bar, 50 µm). **b**, Expression of flocculation-related genes (*FLO1* or *FLO5*) in Cell 1 (007Δ *FLO1 sfGFP,* Green) and Cell 2 (*007Δ FLO1 mRuby2,* Red) results in strong but non-specific adhesion. **c**, Specific adhesion pairs (Strategy 2) expressed in Cell 1 (007Δ *SAG1 sfGFP,* Green) and Cell 2 (007Δ *AGA1 AGA2 mRuby2,* Red) lead to targeted cell-cell interactions. Strategy 2.1. was selected as an example of Strategy 2 and the other showed a similar pattern and can be found in **Supplementary Fig. 3f**. **d**, Proportion analysis of cell adhesion types across different adhesion systems (Strategy 1, Strategy 2.1 and Strategy 2.2), with percentages indicated on bars. Multicellular with Red and Green: Cell clusters of more than three cells containing both Cell 1 (Green) and Cell 2 (Red); Multicellular with Red or Green: Cell lusters of more than three cells containing either Cell 1 (Green) or Cell 2 (Red) exclusively; Tri-/Bicellular with Red and Green: Cell clusters of two or three cells containing both Cell 1 (Green) and Cell 2 (Red); Tri-/Bicellular with Red or Green: Cell clusters of two or three cells containing either Cell 1 (Green) or Cell 2 (Red) exclusively. Quantification was determined by calculating the ratio of each cell type to the total cell count, derived from six confocal images obtained from confocal sections of three replicate samples. **e**, Specific adhesion pairs (Strategy 2.2) mediate adhesion between Cell 1 and Cell 2, but not to Cell 3. Microscopy images (Strains): 007Δ *Ag3-FLO5C sfGFP* (Green cells), 007Δ *Nb3-FLO5 mRuby2*

(Red cells)*,* 007Δ *mTagBFP2* (Blue cells). **f**, Specific adhesion pairs (Strategy 2.2) expressed in Cell 1 and Cell 2 (007Δ *AGA1 AGA2 mRuby2,* Red) lead to targeted cell-cell interactions. Microscopy images (Strains): 007Δ *Ag3-FLO5C sfGFP* (Green cells), 007Δ *Nb3-FLO5 mRuby2* (Red cells). Figures present representative fluorescent images from experiments that were independently repeated at least three times with similar outcomes.

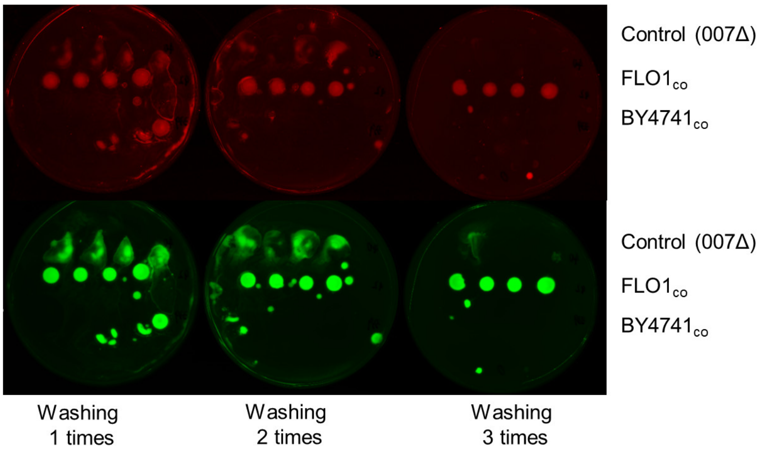

Supplementary Fig. 4 Two engineered cells (Cell 1 and Cell 2) were used for assessing their ability to form 2D patterns.

Cell 1 was first plated on SM agar and cultured for three days. Subsequently, Cell 2 (OD_{600nm} = 20) was overlaid on Cell 1 and incubated for 30 minutes before being gently washed three times with SM medium. All images were acquired using a Typhoon biomolecular imager. Control conditions included $BY4741_{co}$ (Cell 1: BY4741 *sfGFP*, Cell 2: BY4741 *mRuby2*) and the non-flocculating mutant (Cell 1: 007Δ *sfGFP*, Cell 2: 007Δ *mRuby2*). The $FLO1_{co}$ co-culture (Cell 1: 007Δ *FLO1 sfGFP*, Cell 2: 007Δ *FLO1 mRuby2*) was used to highlight the impact of *FLO1* expression on cell adhesion and pattern formation.

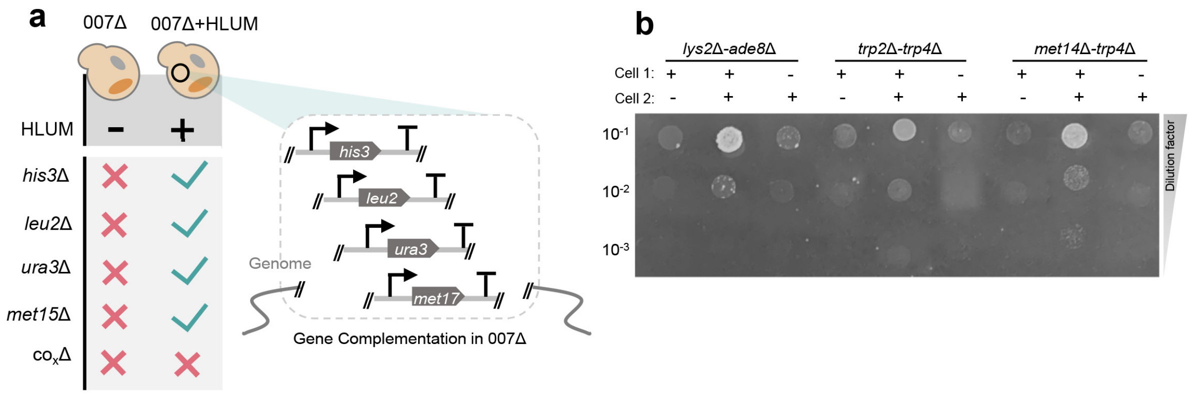

Supplementary Fig. 5 Colony growth assays reveal cross-feeding interactions and auxotrophic requirements in synthetic yeast co-cultures.

a, To assess the effects of specific auxotrophic mutations, 007Δ-derived deletion strains were complemented with plasmids carrying the *His3*, *Leu2*, *Ura3*, and *Met17* genes (HLUM). As these complemented strains no longer require histidine, leucine, methionine, or uracil for growth in synthetic minimal (SM) medium, any observed growth deficiencies can be attributed to the remaining auxotrophic mutations (*lys2*Δ, *ade8*Δ, *trp2*Δ, *trp4*Δ and *met14*Δ). **b,** Colony growth on SM agar for three cross-feeding co-cultures with varying cell combinations and dilutions (10, 100, 1000-fold) from an initial OD_{600nm} of 2.0. Each spot represents 10 µL of inoculum. "-" indicates replacement with an equal volume of SM medium. Cells were inoculated at a 1:1 ratio and incubated for 4 days.

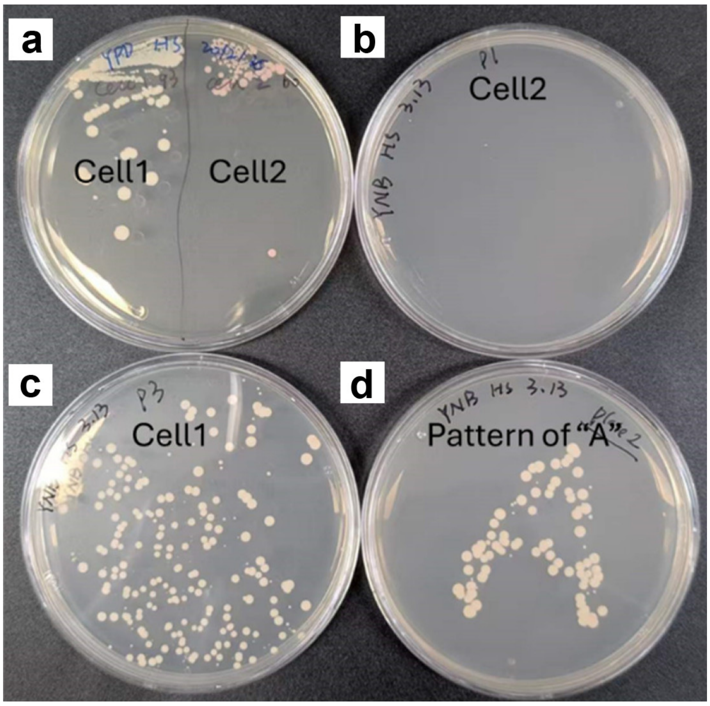

Supplementary Fig. 6 Cell patterning test.

a, Prototrophic Cell 1 and auxotrophic Cell 2 grew on YPD agar. **b,** Auxotrophic Cell 2 did not grow on the synthetic minimal (SM) agar due to adenine auxotroph. **c,** Prototrophic Cell 1 showed growth in SM agar. **d,** An "A" pattern was formed with prototrophic Cell 1 on SM agar.

The patterning test protocol was as follows. Auxotrophic Cell 1: *BY4741-pHLUM*, Prototrophic Cell 2: *BY4741 ADE8△-pTDH3-mScarlet-I-tADH1-vLEU2-pHUM*.

1. Seed cultures of Cell 1 and Cell 2 were inoculated from fresh agar plates (Plate A) into YPD liquid medium and grown overnight.
2. Cells were washed twice with SM medium and diluted to an OD_{600nm} 0.1 using the Implen OD600 spectrophotometer (equivalent to the initial OD_{600nm} of 0.2 described in the Methods, as measured by the Biochrom WPA Lightwave II).
3. 150 µL of Cell 2 was spread onto SM agar plates B & D, which were then left in a biosafety cabinet for approximately 30 minutes until fully dried.
4. 150 µL of Cell 1 was spread onto SM agar plate C. An "A" pattern was drawn on SM agar plate D using Cell 2 with a 10 µL inoculation loop.
5. Put all three plates in 30°C for 3-4 days and take photos.

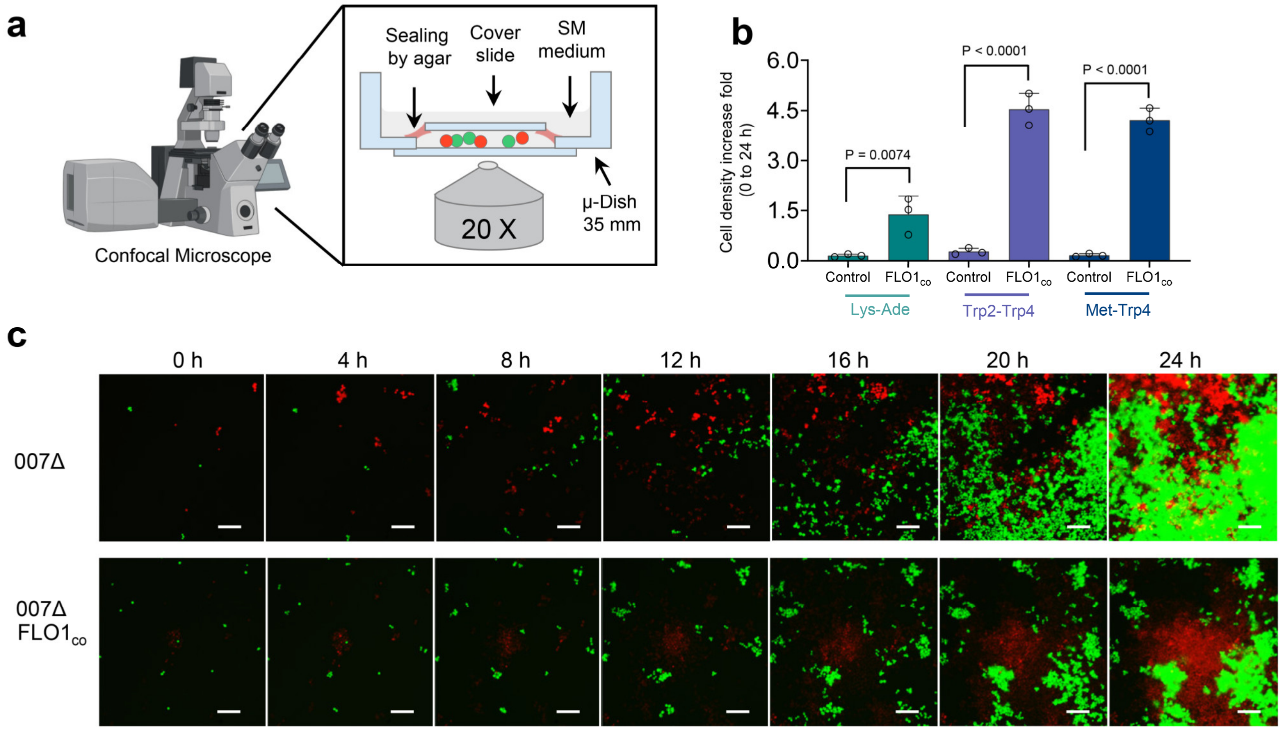

Supplementary Fig. 7 Time-lapse microscopy reveals dynamic cell growth and patterning in synthetic yeast co-cultures with engineered adhesion.

a, Schematic diagram of the time-lapse microscopy setup for yeast co-cultures. A 20 μL aliquot of mixed cell solution (Cell 1 + Cell 2; OD_{700nm} = 0.4) was carefully placed on a 35 mm μ-Dish, covered with a coverslip, and sealed using 1% low-melting-point agar. Time-lapse imaging was performed using a Nikon Eclipse Ti microscope with a 20x S Plan Fluor ELWD DIC N1 objective lens. Cells were incubated at 30°C for 36 h (007Δ for 24 h). The schematic was created with BioRender.com. **b**, Quantification of cell fold increase after introducing FLO1 in Lys-Ade, Trp2-Trp4, and Met-Trp4 cross-feeding co-cultures from 0 to 24 hours in time-lapse microscopy observations. *n* = 3 biologically independent samples and data are presented as mean ± s.d.. Statistical analysis was executed using Prism 9.5.0 (GraphPad) software with one-way ANOVA, followed by Tukey's post-hoc test and P values were noted. **c**, Time-lapse confocal fluorescence microscopy images of control groups for three synthetic co-cultures exhibiting cross-feeding and adhesion. Cell growth and pattern formation are shown for the 007Δ and 007Δ *FLO1* strains co-culture. Scale bar, 50 μm. See also **Supplementary Videos 7-8**.

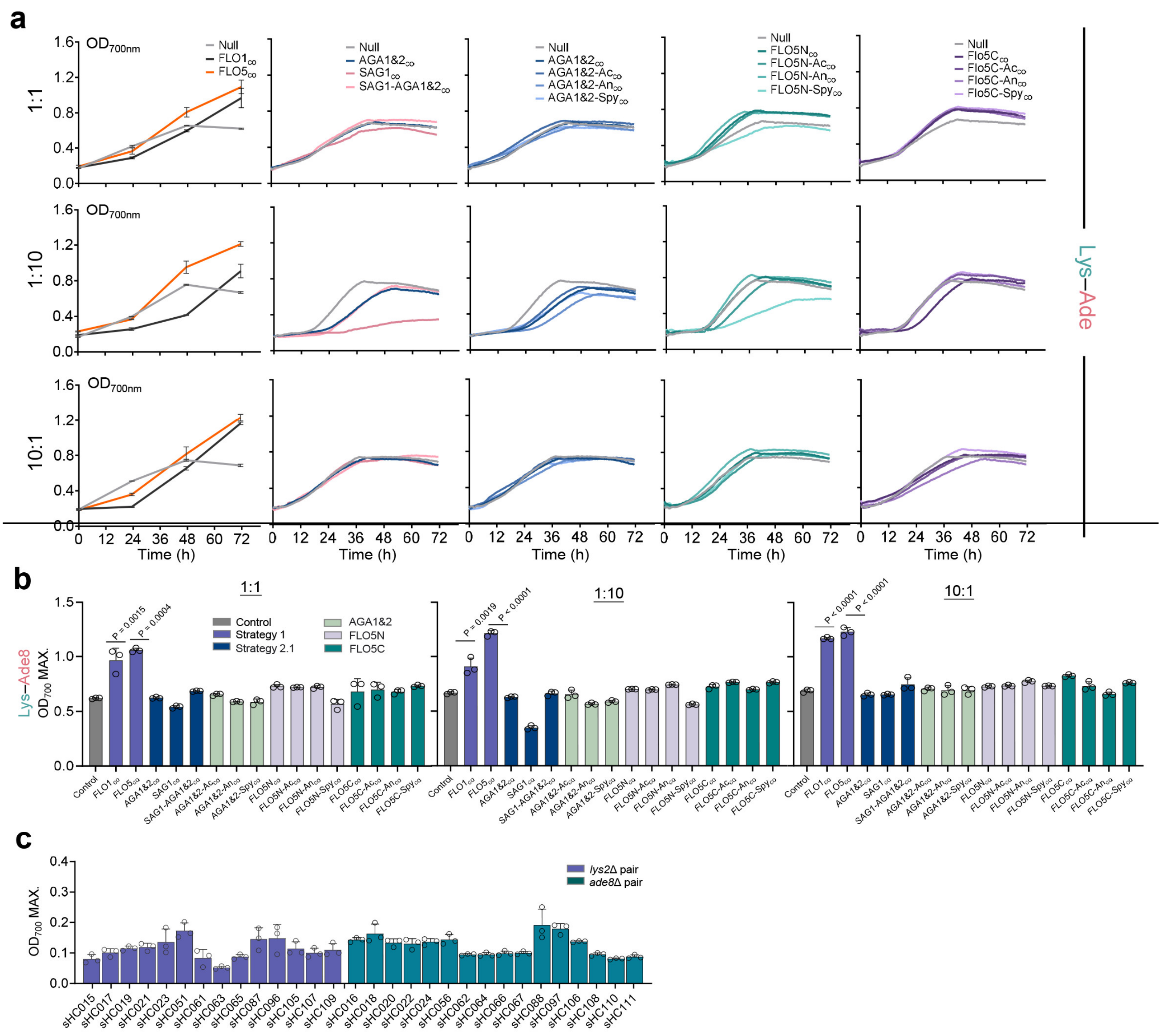

Supplementary Fig. 8 Impact of cross-feeding pairs and inoculation ratios on the growth dynamics of Lys-Ade co-cultures with engineered adhesion.

a, The Lys-Ade cross-feeding pair was co-cultured with each of the 16 adhesion pairs at three different inoculation ratios (1:1, 1:10, and 10:1). Growth (OD_{700nm}) was monitored continuously over 72 h, except for $FLO1_{co}$ and $FLO5_{co}$ co-cultures, which exhibited strong flocculation and were measured only at 0, 24, 48, and 72 h. **b**, Maximal OD_{700} values of Lys-Ade co-cultures and controls of monocultures within 72 h. In these two-member co-cultures, the initial ratios were 1:1, 1:10 and 10:1, respectively. **c**, Maximal OD_{700} values of negative controls of auxotrophic monocultures used for Lys-Ade co-cultures. n = 3 biologically independent samples and data are presented as mean ± s.d.. Statistical analysis was executed using Prism 9.5.0 (GraphPad) software with one-way ANOVA, followed by Tukey's post-hoc test.

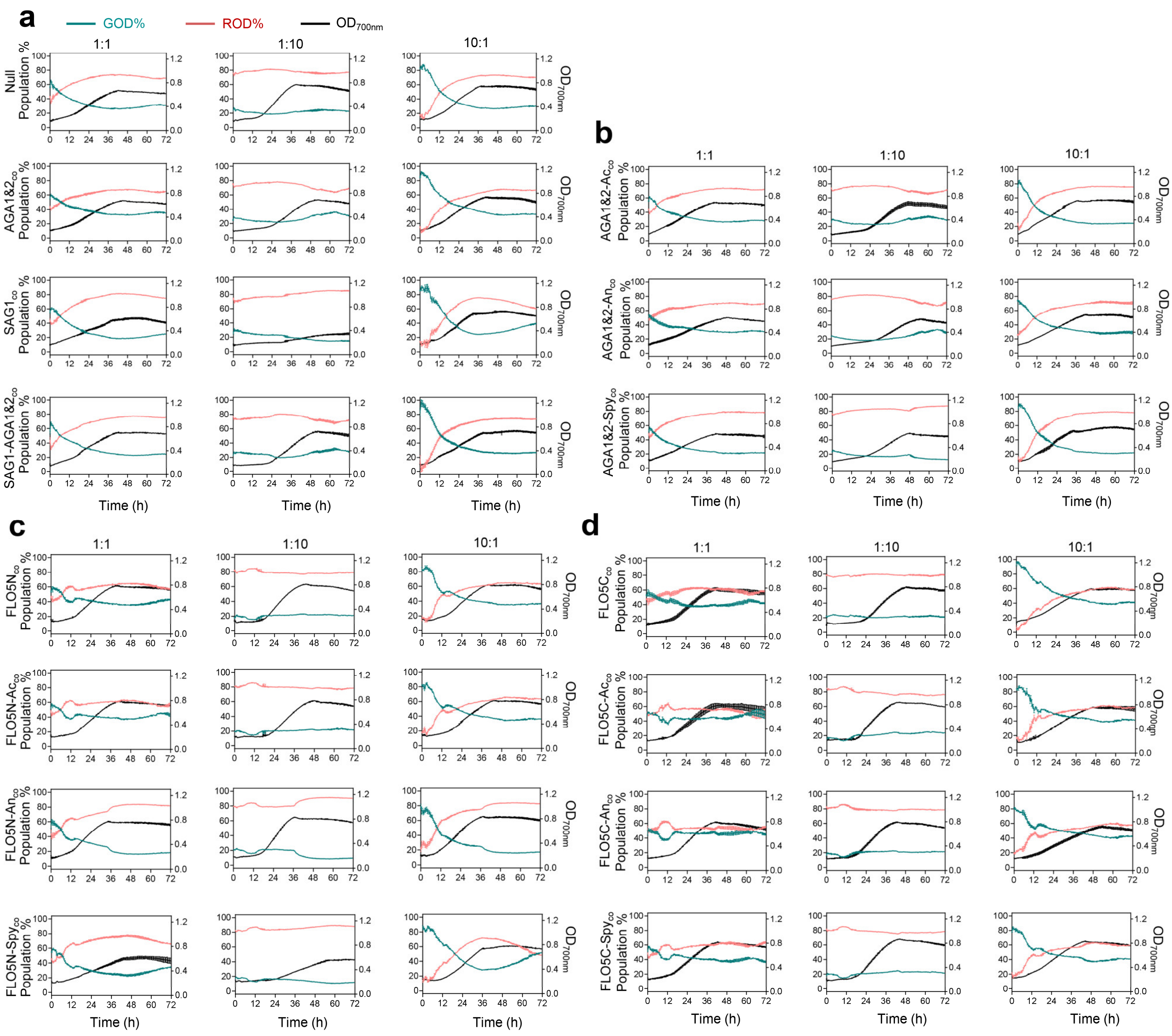

Supplementary Fig. 9 Impact of intercellular adhesion pairs on the Lys-Ade co-culture system under dynamic condition.

Auxotrophic cross-feeding Lys-Ade co-cultures were combined with 14 adhesion pairs (**a,** Strategy 2.1; **b,** AGA1&2 system; **c,** FLO5N system; **d,** FLO5C system), and inoculated at three different ratios (1:1, 1:10, and 10:1). Cultures were incubated at 30℃ with shaking (250 rmp). Total co-culture density (OD_{700nm}, black line), estimated OD_{700nm} of the sfGFP-tagged population percentages (GOD%, green line), and estimated OD_{700nm} of the mRuby2-tagged population percentages (ROD%, red line) were monitored. n = 3 biologically independent samples and data are presented as mean ± s.d..

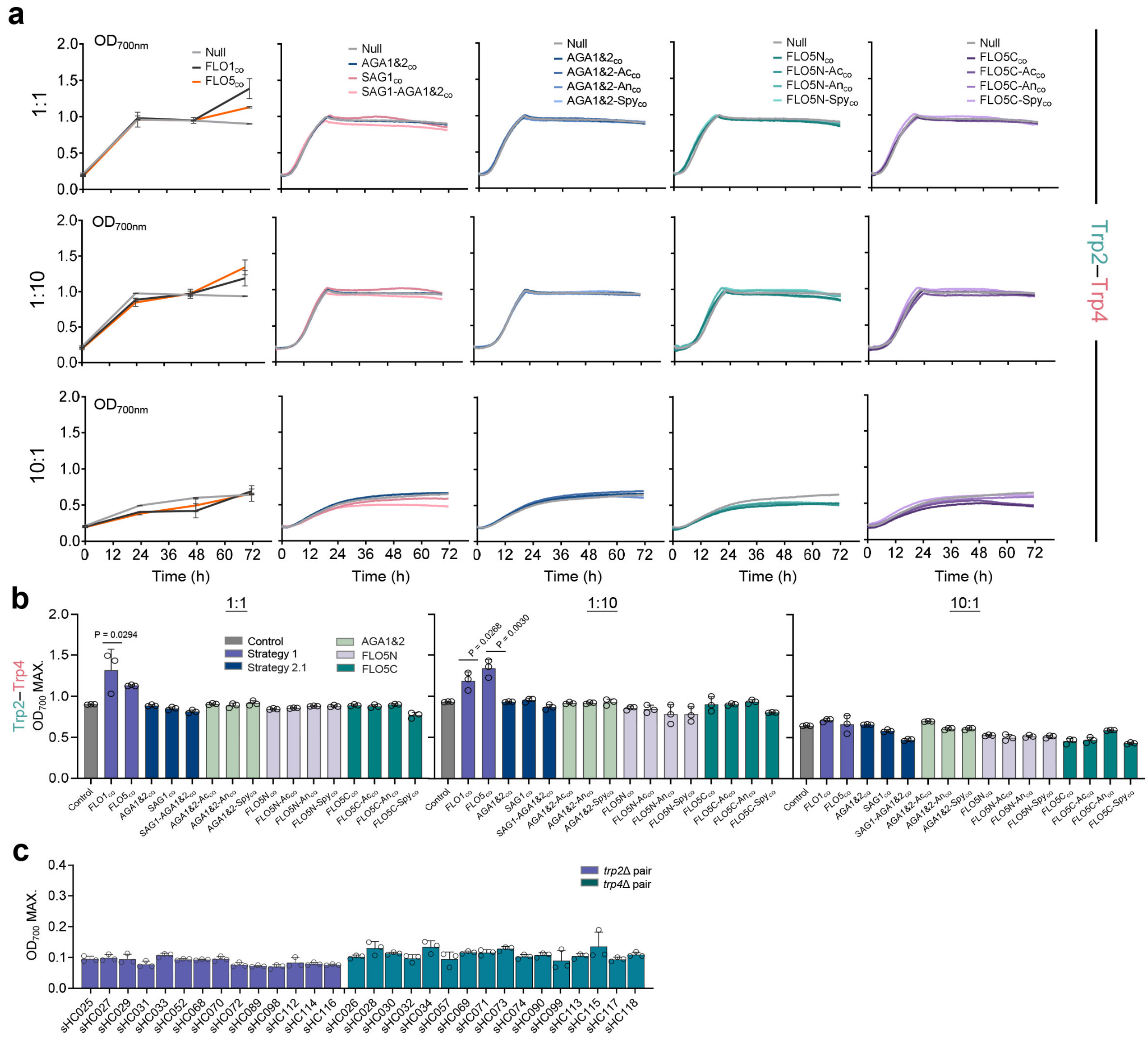

Supplementary Fig. 10 Impact of cross-feeding pairs and inoculation ratios on the growth dynamics of Trp2-Trp4 co-cultures with engineered adhesion.

a, The Trp2-Trp4 cross-feeding pair was co-cultured with each of the 16 adhesion pairs at three different inoculation ratios (1:1, 1:10, and 10:1). Growth (OD_{700nm}) was monitored continuously over 72 h, except for $FLO1_{co}$ and $FLO5_{co}$ co-cultures, which exhibited strong flocculation and were measured only at 0, 24, 48, and 72 h. **b**, Maximal OD_{700nm} values of Trp2-Trp4 co-cultures and controls of monocultures within 72 h. In these two-member co-cultures, the initial ratios were 1:1, 1:10 and 10:1, respectively. **c**, Maximal OD_{700nm} values of negative controls of auxotrophic monocultures used for Trp2-Trp4 co-cultures. *n* = 3 biologically independent samples and data are presented as mean ± s.d.. Statistical analysis was executed using Prism 9.5.0 (GraphPad) software with one-way ANOVA, followed by Tukey's post-hoc test.

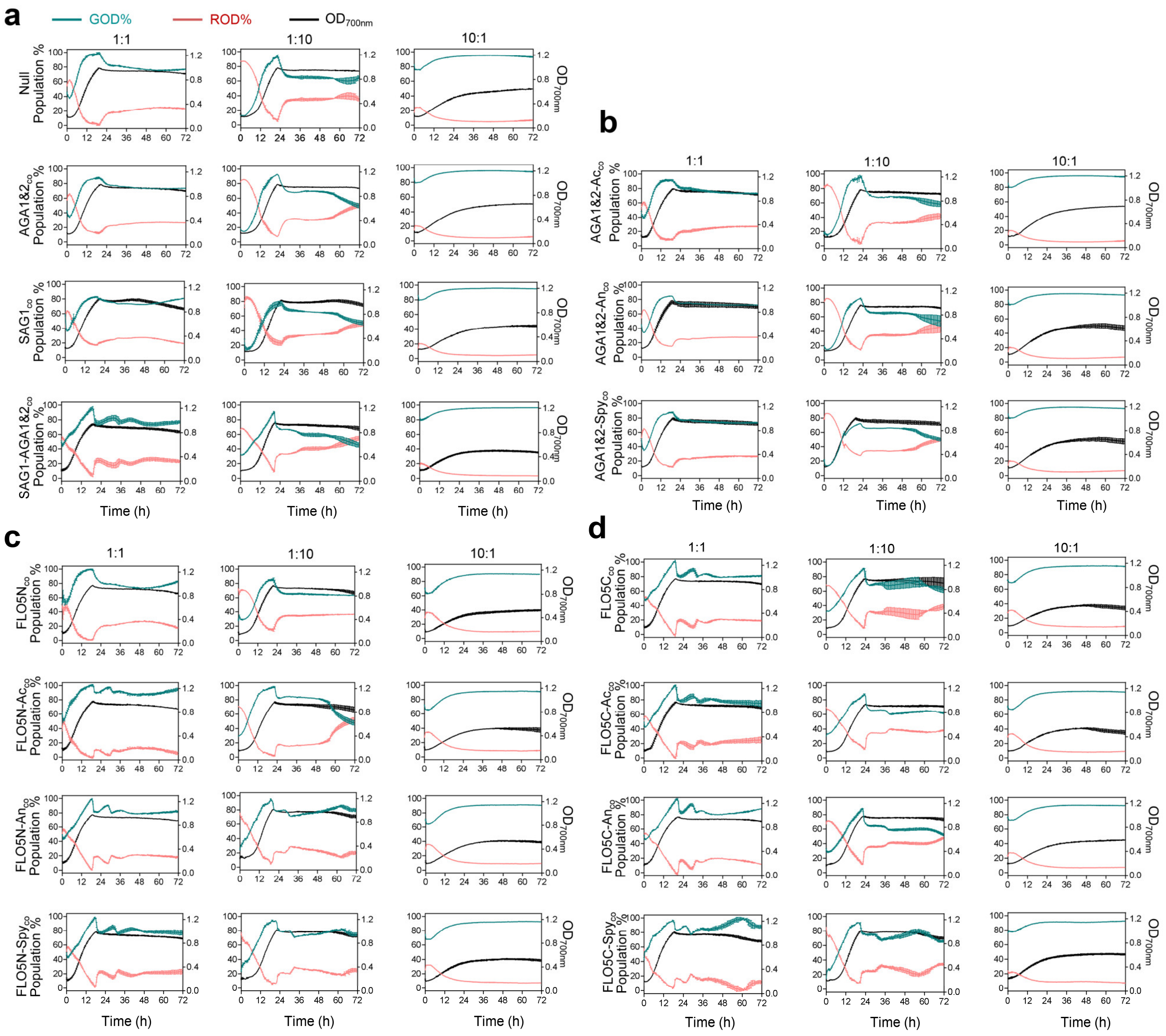

Supplementary Fig. 11 Impact of intercellular adhesion pairs on the Trp2-Trp4 co-culture system under dynamic condition.

Auxotrophic cross-feeding Trp2-Trp4 co-cultures were combined with 14 adhesion pairs (**a,** Strategy 2.1; **b,** AGA1&2 system; **c,** FLO5N system; **d,** FLO5C system), and inoculated at three different ratios (1:1, 1:10, and 10:1). Cultures were incubated at 30℃ with shaking (250 rmp). Total co-culture density (OD_{700nm}, black line), estimated OD_{700nm} of the sfGFP-tagged population percentages (GOD%, green line), and estimated OD_{700nm} of the mRuby2-tagged population percentages (ROD%, red line) were monitored. *n* = 3 biologically independent samples and data are presented as mean ± s.d..

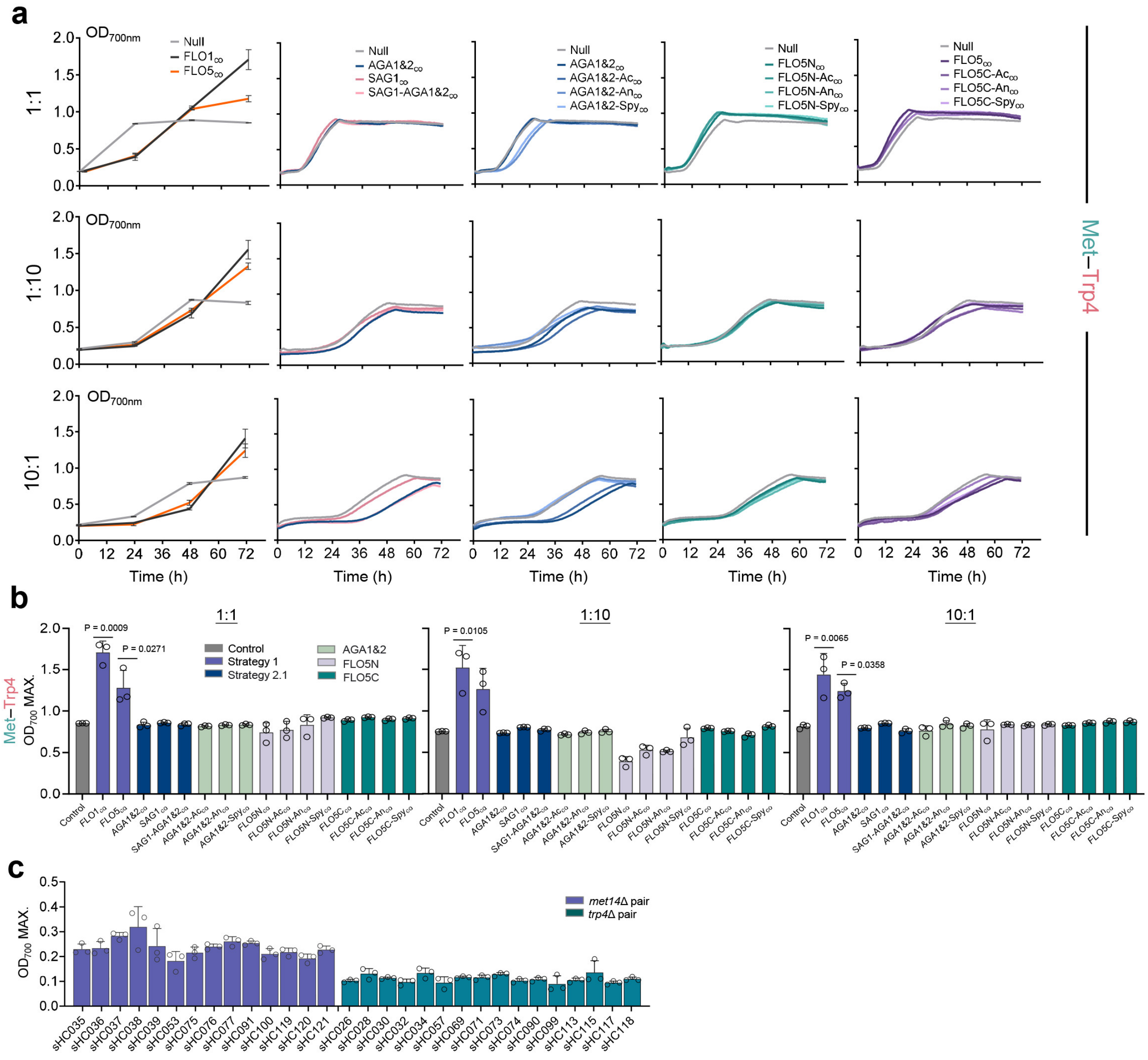

Supplementary Fig. 12 Impact of cross-feeding pairs and inoculation ratios on the growth dynamics of Met-Trp4 co-cultures with engineered adhesion.

a, The Met-Trp4 cross-feeding pair was co-cultured with each of the 16 adhesion pairs at three different inoculation ratios (1:1, 1:10, and 10:1). Growth (OD_{700nm}) was monitored continuously over 72 h, except for $FLO1_{co}$ and $FLO5_{co}$ co-cultures, which exhibited strong flocculation and were measured only at 0, 24, 48, and 72 h. **b**, Maximal OD_{700nm} values of Met-Trp4 co-cultures and controls of monocultures within 72 h. In these two-member co-cultures, the initial ratios were 1:1, 1:10 and 10:1, respectively. **c**, Maximal OD_{700nm} values of negative controls of auxotrophic monocultures used for Met-Trp4 co-cultures. n = 3 biologically independent samples and data are presented as mean ± s.d.. Statistical analysis was executed using Prism 9.5.0 (GraphPad) software with one-way ANOVA, followed by Tukey's post-hoc test.

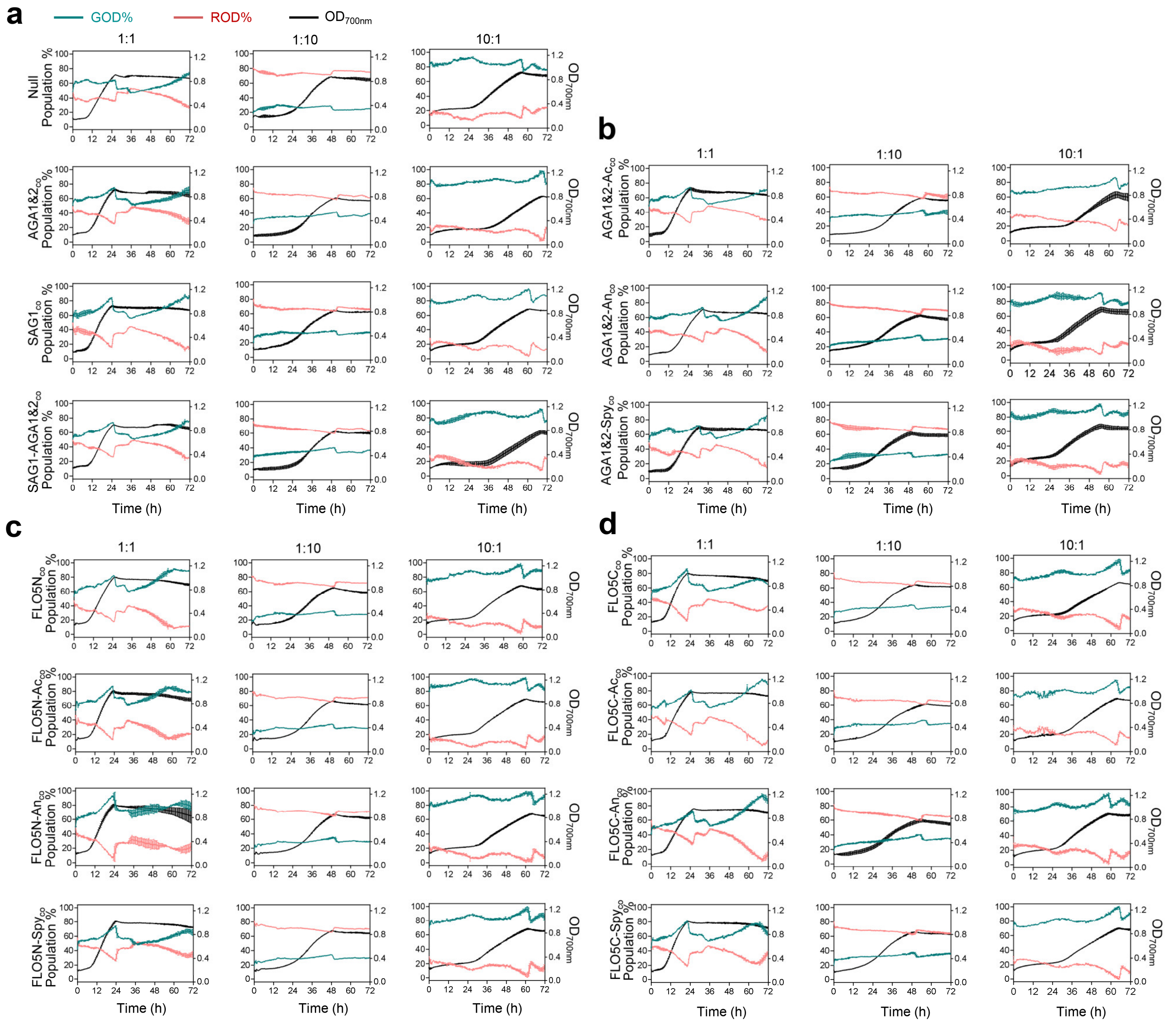

Supplementary Fig. 13 Impact of intercellular adhesion pairs on the Met-Trp4 co-culture system under dynamic condition.

Auxotrophic cross-feeding Met-Trp4 co-cultures were combined with 14 adhesion pairs (**a,** Strategy 2.1; **b,** AGA1&2 system; **c,** FLO5N system; **d,** FLO5C system), and inoculated at three different ratios (1:1, 1:10, and 10:1). Cultures were incubated at 30°C with shaking (250 rmp). Total co-culture density (OD_{700nm}, black line), estimated OD_{700nm} of the sfGFP-tagged population percentages (GOD%, green line), and estimated OD_{700nm} of the mRuby2-tagged population percentages (ROD%, red line) were monitored. n = 3 biologically independent samples and data are presented as mean ± s.d..
